# Selection on Network Dynamics Drives Differential Rates of Protein Domain Evolution

**DOI:** 10.1371/journal.pgen.1006132

**Published:** 2016-07-05

**Authors:** Brian K. Mannakee, Ryan N. Gutenkunst

**Affiliations:** 1 Division of Epidemiology and Biostatistics, Mel and Enid Zuckerman College of Public Health, University of Arizona, Tucson, Arizona, United States of America; 2 Department of Molecular and Cellular Biology, University of Arizona, Tucson, Arizona, United States of America; University of Michigan, UNITED STATES

## Abstract

The long-held principle that functionally important proteins evolve slowly has recently been challenged by studies in mice and yeast showing that the severity of a protein knockout only weakly predicts that protein’s rate of evolution. However, the relevance of these studies to evolutionary changes within proteins is unknown, because amino acid substitutions, unlike knockouts, often only slightly perturb protein activity. To quantify the phenotypic effect of small biochemical perturbations, we developed an approach to use computational systems biology models to measure the influence of individual reaction rate constants on network dynamics. We show that this dynamical influence is predictive of protein domain evolutionary rate within networks in vertebrates and yeast, even after controlling for expression level and breadth, network topology, and knockout effect. Thus, our results not only demonstrate the importance of protein domain function in determining evolutionary rate, but also the power of systems biology modeling to uncover unanticipated evolutionary forces.

## Author Summary

Different proteins evolve at dramatically different rates. To understand this variation, it is necessary to determine which characteristics of proteins are visible to natural selection and how the strength of selection depends on those characteristics. One protein characteristic that is evidently visible to natural selection is expression level; more highly expressed proteins are subject to stronger purifying selection and evolve more slowly. Theory and intuition suggest another such characteristic should be some measure of functional importance, but studies of various measures of functional importance, such as knockout essentiality or knockout growth rate, have shown at best weak correlations with evolutionary rate. Here we develop a novel measure of functional importance, *dynamical influence*, which quantifies the importance of a protein or protein domain to the dynamics of the network of proteins in which it functions. Using 18 biochemically-detailed systems biology models, we compute dynamical influences for each protein domain in each model. We find that dynamical influence is indeed visible to natural selection and that within networks protein domains with higher dynamical influence evolve more slowly.

## Introduction

Over evolutionary time, every protein accumulates amino acid changes at its own characteristic rate, which Zuckerkandl and Pauling likened to the ticking of a molecular clock [1]. Remarkably, this evolutionary rate varies by orders of magnitude among proteins. Understanding the determinants of this variation is a fundamental goal in molecular evolution research [2–5]. Early theoretical work suggested that functional constraints within proteins [1] and the functional importance of each protein to the organism [6, 7] would be key factors in determining evolutionary rates. Yet, empirical studies using knockouts have observed only weak effects. In bacteria [8, 9], yeast [10, 11], and mammals [12] knockout studies conclude that essential proteins evolve only slightly more slowly than non-essential proteins. Moreover, among non-essential genes in yeast, there is little to no correlation between the effect of a protein knockout on growth rate, in a wide range of conditions, and that protein’s evolutionary rate [11, 13, 14], particularly when controlling for expression level [15]. This poor correlation between knockout effects and rates of protein evolution has led some researchers to conclude that function-specific selection plays little role in determining evolutionary rates [4, 5]. This conclusion, however, contradicts theoretical expectations, the intuition of most molecular biologists, and the reasoning behind much of comparative genomics [16], motivating our search for an alternative measure of protein function.

**Fig 1:**
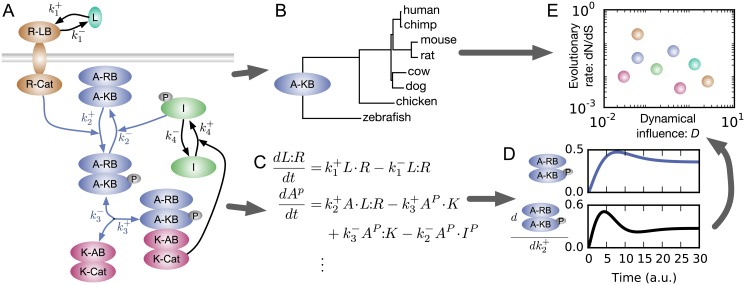
Overview of analysis. A: Illustrative hypothetical signaling network. The dynamical influence of the activator kinase-binding domain (A-KB) is calculated from the influences of the rate constants of the reactions in which it is involved (highlighted in blue): phosphorylation, $k_{2}^{+}$; dephosphorylation, $k_{2}^{-}$; kinase-binding, $k_{3}^{+}$; and kinase-unbinding, $k_{3}^{-}$. B: Illustrative phylogenetic analysis of the kinase-binding domain of the activator protein. C: Partial list of ordinary differential equations that model the dynamics of this network. Here all reactions are assumed to be mass-action, but that is not the case in all models analyzed. D: Dynamics of phosphorylated activator protein levels and sensitivity of those dynamics to changes in rate constant $k_{2}^{+}$, following addition of ligand L. Small increases in $k_{2}^{+}$ hasten the peak of phosphorylated activator protein and increase its steady-state level. The dynamical influence of rate constant $k_{2}^{+}$ is calculated by summing such sensitivities for all molecular species in the network. E: Illustrative plot comparing dynamical influence and evolutionary rate for all domains in the network. A single multi-domain protein can contribute multiple data points.

We reasoned that knockouts do not mimic evolutionarily relevant mutations, which often have small or moderate effects [17]. In particular, most amino-acid changes do not completely destroy a protein’s function, but rather alter its biochemical activity to a greater or lesser extent [18]. The ideal experiment would thus measure the functional effects of many random mutations on many proteins, but such experiments remain challenging [19]. To overcome this experimental limitation, we undertook a computational approach, using biochemically-detailed systems biology models to predict the effects that small perturbations to protein activities will have on the dynamics of the networks in which they function (Fig 1). We ascribed high and low *dynamical influence* to protein domains for which amino acid substitutions were predicted to have respectively large or small effects on network dynamics. We hypothesized that network dynamics is a synthetic phenotype that is likely subject to natural selection. To test this hypothesis, we compared our predictions of dynamical influence in functionally and structurally conserved intracellular signaling and biosynthetic networks with genomic data on protein domain evolutionary rates in both vertebrates and yeast. We found that, within these networks, dynamical influence is as strongly correlated with evolutionary rate as many previously known correlates. Moreover, dynamical influence remains predictive when knockout phenotype, expression, and network topology are controlled for. Dynamical influence thus offers new insight into selective constraint within dynamical protein networks.

## Results and Discussion

### Dynamical influence quantifies the network consequences of small-effect mutations

Biochemically-detailed systems biology models encapsulate vast amounts of molecular biology knowledge in a form that can be used for *in silico* experimentation [20, 21]. In particular, they enable simulation of the dynamics of molecular species (e.g., proteins, metabolites, modified forms, and complexes) concentrations under a variety of conditions. In these models, protein biochemical activities are quantified by reaction rate constants *k* [22]. To assess the phenotypic effects of small changes in protein activity caused by mutations, we first calculated the dynamical influence of each reaction rate constant (Eq 1, Materials and Methods). To do so, we calculated how a differential perturbation to that constant would change the concentration time course of each molecular species in the network (Fig 1D), for biologically-relevant stimuli. We then normalized those changes and integrated the squared changes over time. Lastly, we summed over all molecular species in the network. The dynamical influence of a rate constant is thus the total effect that small changes in that rate constant would have on network dynamics.

The dynamical influence of each reaction rate constant quantifies its importance to network dynamics, but there is little data on evolutionary divergence of reaction rate constants to which we can compare. To compare with the abundant genomic data detailing sequence divergence at the domain level, we aggregated the influences of reaction rate constants for all reactions in which a given protein domain is involved. Whenever possible, we analyzed at the domain level, because that is the level at which distinct functions can be assigned to distinct regions of protein sequence [23]. Thus, we defined the dynamical influence *D* of a domain to be the geometric mean of the dynamical influences of the reaction rate constants for reactions in which it participates (Fig 1A, Eq 2). In general, any mutation in a domain will cause a multidimensional perturbation of all rate constants associated with that domain. Furthermore, different mutations in a domain will differ in the overall magnitude of that perturbation and its relative effect on different parameters [24]. Unfortunately, little systematic data exists about the distributions of such perturbations. The geometric average we took is an approximation to the more complex averaging that occurs as various mutations arise over evolutionary time. As more systematic data is generated about mutation effects on biochemical activities of different domains [19], the geometric average may be replaced by domain-specific distributions of perturbations.

### Dynamical influence within networks is correlated with protein domain evolutionary rate

To test whether dynamical influence is informative about protein evolution, we analyzed dynamic protein network models from BioModels [25], a database which not only collects such models but also annotates them with links to other bioinformatic databases [26, 27]. We considered only models with experimental validation that were formulated in terms of molecular species and reactions, were runnable as ordinary differential equations, and contained at least eight distinct UniProt protein annotations. In total, we studied 12 vertebrate [28–39] and 6 yeast [40–45] signaling and biosynthesis models. We further annotated these models to connect molecular species and reactions with particular protein domains (S1 Dataset). For each model, we calculated dynamical influences for each reaction rate constant using the stimulation conditions considered in the model’s original publication (S1 Text).

**Fig 2:**
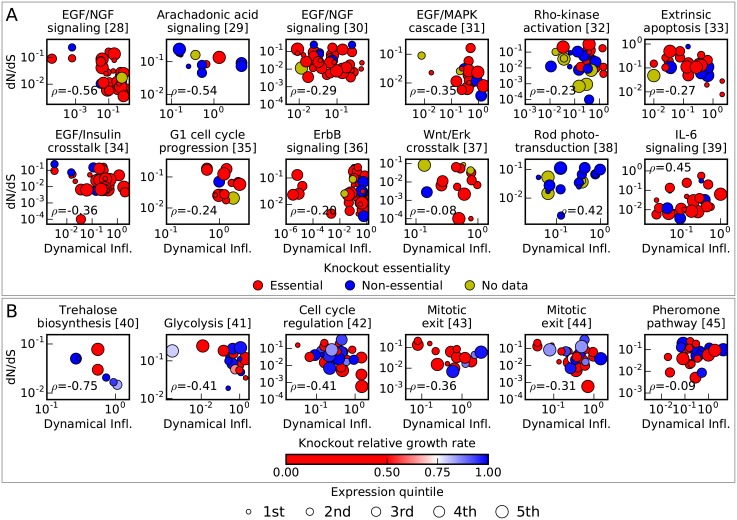
Domain evolutionary rate is correlated with dynamical influence in signaling and biosynthetic networks. Each point represents a protein domain, plotted given its evolutionary rate dN/dS and dynamical influence. Spearman rank correlations *ρ* between dynamical influence and evolutionary rate are generally negative, indicative of widespread purifying selection on network dynamics. Expression level is represented by marker size and is weakly correlated with evolutionary rate but not significantly correlated with dynamical influence (Table 1). A: Vertebrate networks. Knockout essentiality is represented by color and is not significantly correlated with evolutionary rate or dynamical influence (Table 1). B: Yeast networks. Knockout growth rate is represented by color, with red indicating a more severe phenotype. Knockout growth rate is not significantly correlated with evolutionary rate or dynamical influence (Table 1).

Using this novel method, we determined protein domain dynamical influence and evolutionary rate for 18 conserved signaling and metabolic networks (Fig 2). We quantified the strength of the relationship between dynamical influence and evolutionary rate using Spearman rank correlations (*ρ*), and in 10 of 12 vertebrate networks and 6 of 6 yeast networks, we found a negative correlation. This is consistent with the expectation that most sequences and networks evolve primarily under purifying selection [46], in which natural selection is primarily acting to remove deleterious mutations from the population. Mutations in protein domains with high dynamical influence are predicted to have greater phenotypic effect and thus, in general, be more deleterious. So mutations in those domains are more efficiently removed, and those domains evolve more slowly. Demonstrating the strength of our approach, the two exceptional vertebrate models with a positive correlation, visual signal transduction and interleukin 6 (IL-6) signaling, were recently identified as undergoing network-level adaptation in humans using population genetic data [47]. Positively selected molecular changes in rhodopsin associated with changes in absorption wavelength have been shown to affect dose-response behavior in visual signal transduction [48, 49], suggesting that network-level adaptation may compensate for changes in rhodopsin. As part of the innate immune system, IL-6 and its receptor evolve under strong diversifying selection, so downstream proteins may evolve to maintain signal fidelity. Moreover, viruses are known that directly interfere with proteins downstream of IL-6 [50, 51], potentially driving additional adaptation. Dynamical influence is thus predictive not only about purifying selection but also about adaptive selection.

**Fig 3:**
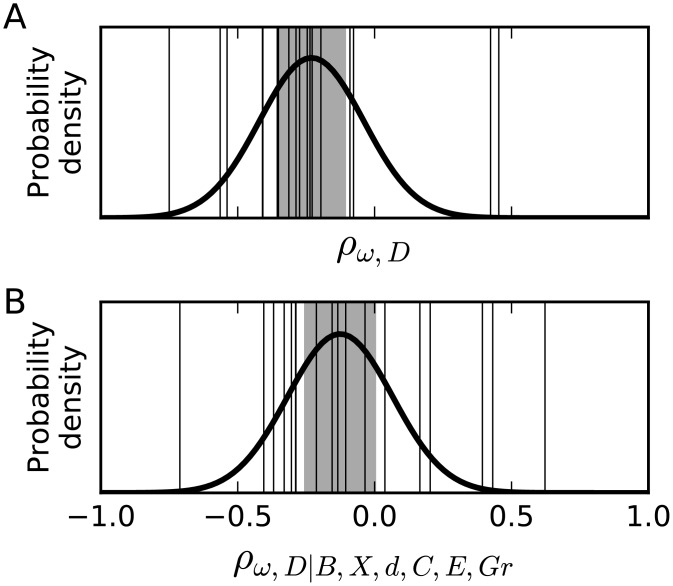
Meta-analysis of correlations between protein domain evolutionary rate and dynamical influence. A: The measured correlation between protein domain evolutionary rate and dynamical influence varies among sampled networks (thin vertical lines). The meta-analysis estimates the distribution of the correlation within the population of networks (thick normal curve), and the shaded region indicates the 95% confidence interval for the mean of that distribution. The mean is significantly less than zero, indicating that evolutionary rate and dynamical influence are negatively correlated in most networks, consistent with predominant purifying selection on network dynamics. B: As in A, but controlling for numerous other factors known to correlated with evolutionary rate, as defined in Table 1.

The strength of the correlation between dynamical influence and protein domain evolutionary rate varies considerably among networks (Fig 3A, S1 and S2 Tables). Dynamical influence quantifies the relative effects of perturbations within a single network. Directly comparing dynamical influences among networks would require assumptions about the heterogenous relative fitness impact of those networks, such as EGF/NGF signaling versus cell cycle progression. Pooling of heterogeneous data can lead to biased estimates of overall correlations [53, 54]. To avoid such bias, we did not pool data across networks but rather applied meta-analysis.

Because selection may act differently on networks with different functions, we considered a random-effects meta-analysis. Thus the sampled networks were assumed to represent a population of networks, among which the correlation between domain evolutionary rate and dynamical influence varies. The meta-analysis seeks to estimate the distribution of those correlations. We applied the random-effects meta-analysis method of Hunter and Schmidt [52], because simulation studies suggest that it provides an accurate estimate of the mean correlation, particularly when that correlation is modest [55, 56]. The estimated distribution of correlations between domain evolutionary rate and dynamical influence is wide, but the 95% confidence interval for the mean correlation excludes zero (Fig 3A and Table 1). This suggests that negative correlation is more common than positive correlation, consistent with the expectation that purifying selection is more common than adaptive selection [46].

**Table 1:** Overall correlations between domain evolutionary rate, dynamical influence, and other explanatory variables. Spearman rank (*ρ*) and rank biserial (*rb*) correlation coefficients for variables evolutionary rate dN/dS (*ω*), dynamical influence (*D*), expression breadth (*B*, for vertebrates only), expression level (*X*), interaction degree (*d*), interaction betweenness centrality (*C*), knockout essentiality (*E*), and knockout growth rate (*Gr*, for yeast only). Mean population correlations and their confidence intervals were estimated from all analyzed models using the random-effects meta-analysis approach of Hunter and Schmidt [52]. For a complementary test of the null hypothesis of independence between variables, two-sided p-values were calculated via permutation (Materials and Methods). Dynamical influence is independently predictive of evolutionary rate, as shown by the negative and statistically significant mean partial correlation after controlling for all other variables.

	estimated population mean correlation (95% C.I.)	permutation p-value
*ρ*_*ω*,*D*_	-0.23 (-0.35, -0.11)	<0.0001
*ρ*_*ω*,*B*_	-0.25 (-0.34, -0.15)	0.0011
*ρ*_*ω*,*X*_	-0.14 (-0.29, -0.00)	0.0191
*ρ*_*ω*,*d*_	-0.21 (-0.31, -0.11)	0.0004
*ρ*_*ω*,*C*_	-0.18 (-0.26, -0.10)	0.0024
*rb*_*ω*,*E*_	-0.12 (-0.28, +0.04)	0.2064
*ρ*_*ω*,*Gr*_	-0.02 (-0.15, +0.11)	0.8528
*ρ*_*D*,*B*_	+0.17 (+0.03, +0.31)	0.0076
*ρ*_*D*,*X*_	+0.09 (-0.03, +0.21)	0.0838
*ρ*_*D*,*d*_	+0.07 (-0.02, +0.16)	0.1667
*ρ*_*D*,*C*_	+0.06 (-0.04, +0.15)	0.2789
*rb*_*D*,*E*_	+0.08 (-0.13, +0.29)	0.3498
*ρ*_*D*,*Gr*_	+0.11 (-0.10, +0.31)	0.2475
*ρ*_*ω*,*D*|*B*,*X*,*d*,*C*,*E*,*Gr*_	-0.13 (-0.25, +0.00)	0.0125

As a complementary approach to evaluating the relationship between domain evolutionary rate and dynamical influence, we also performed a permutation test for dependence between them. In this test, we compared the estimated mean correlation from the real data with a null distribution of mean correlations calculated from scrambled data (Materials and Methods). This test rejected the null hypothesis that domain evolutionary rate and dynamical influence are independent within networks, consistent with the confidence interval analysis that excludes zero correlation (Table 1).

### Dynamical influence calculation is robust to modeling uncertainties

**Fig 4:**
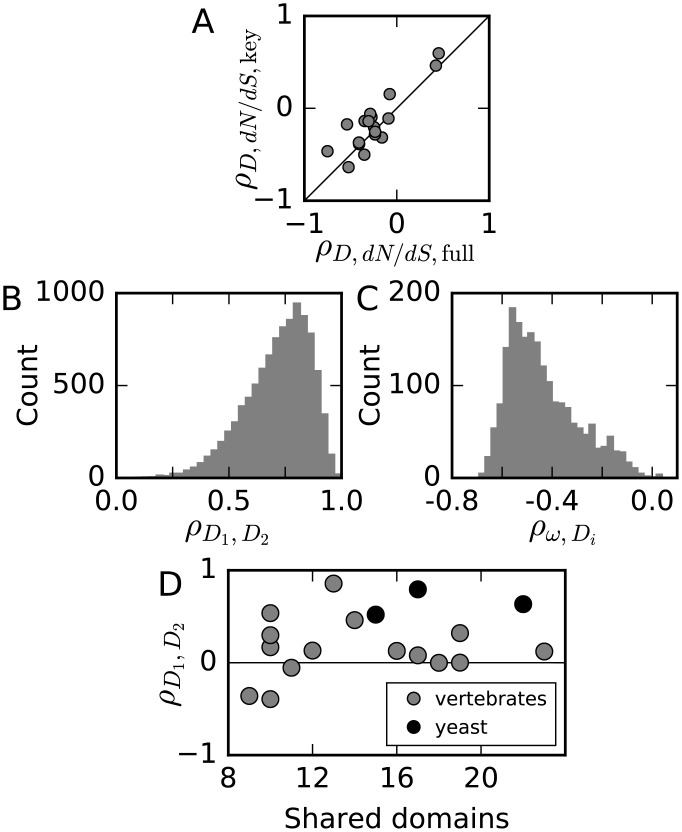
Effects of model uncertainty on dynamical influence. A: The correlation between dynamical influence and evolutionary rate (dN/dS) is similarly strong in all models if dynamical influence is evaluated using all model molecular species (“full”) or only those deemed most important by the authors of the original study (“key”). B: Dynamical influences are strongly correlated between biologically-plausible rate constant sets for a model of EGF/NGF signaling [28]. C: For that model and ensemble of rate constant sets, the correlation between dynamical influence and evolutionary rate varies in magnitude but is consistent in sign. D: Models with overlapping domains produce positively rank-correlated estimates of dynamical influence.

We measured dynamical influence using hand-built systems biology models; what effect do uncertainties in these models have on our analysis? To be agnostic about what aspects of network dynamics are critical to fitness, in calculating dynamical influence we summed over the integrated sensitivities of all molecular species in the network. It is, however, often evident that the builders of each model had specific molecular species in which they were most interested. If we restricted our dynamical influence calculation to those species (S1 Text), we found very similar correlation with domain evolutionary rate (Fig 4A). Our results are thus not strongly sensitive to which aspects of network function are assumed to be subject to natural selection.

Given a network model, substantial uncertainty can exist about the values of the rate constants *k* [22], because they are difficult to measure directly and are thus often fit to experimental data on network behavior [57, 58]. To account for this rate constant uncertainty, an ensemble of rate constant sets consistent with the experimental data and the model can be built [59, 60], but this has unfortunately been done for only a small number of models. To assess the importance of rate constant uncertainty to our results, we used an ensemble of 2000 sets of rate constants [61] that were previously identified as consistent with experimental data for one of our models of EGF/NGF signaling [28]. This ensemble was built by Markov Chain Monte Carlo sampling of the posterior distribution when fitting the model to data from 14 systems biology experiments. Rate constant values in the resulting ensemble vary dramatically, with many values varying by more than four orders of magnitude, but all sets of rate constants reproduce the experimentally-measured network dynamics. We calculated the dynamical influence of all protein domains in the network using all these sets of rate constants. Comparing 10,000 randomly chosen pairs of sets of dynamical influences to each other, we found that they were highly correlated (Fig 4B), with a median rank correlation of 0.74. Over the ensemble of plausible rate constant sets, the correlation between domain dynamical influence and evolutionary rate varied in magnitude, but 99.8% of rate constant sets yielded a negative correlation (Fig 4C). Together these analyses suggest that, while rate constants themselves vary dramatically over the ensemble for this model, relative dynamical influence varies much less, such that rate constant uncertainty does not affect the sign of the observed correlation, although it may affect its magnitude. We could not build rate constant ensembles for the other models in our analysis without access to the original data used to fit those models, but the universality of the “sloppy” pattern of sensitivities in systems biology models [61, 62] suggests that similar results would be found using rate constant ensembles for the other models in our analysis.

In addition to rate constant uncertainty, different modelers may also make different assumptions when studying the same network regarding forms of interaction, which molecular players to include, or which conditions to consider. We assessed the effect of these assumptions using the models in our data which consider overlapping protein domains. The rank correlation between dynamical influences calculated for the same domains using different models varied considerably and was stronger for pairs of models with larger numbers of overlapping domains (Fig 4D and S3 Table). Weighting correlations from different comparisons as in our meta-analysis, we found a mean correlation of 0.26. For comparison, the correlation between different research groups in measurements of gene expression in log-phase growth of budding yeast is roughly 0.62 [63], while for degree in protein-protein interaction data, the correlation is 0.11 [64]. Thus model uncertainty plays a strong but not dominant role in our analysis, and it is comparable to variables that have previously been found to be informative about evolutionary rate.

We defined dynamical influence in terms of differential perturbations to reaction rate constants (Eq 1), but mutations introduce finite perturbations. Dynamical influence values calculated using finite perturbations of ±25% to rate constants were, however, almost perfectly correlated with values calculated using differential perturbations (S4 Table). Our results thus also apply to mutations of moderate effect.

### Dynamical influence predicts evolutionary rates independently from previously known factors

Dynamical influence captures the phenotypic effect within dynamical networks of small perturbations to protein domain activity, but how does it relate to factors previously linked to evolutionary rate? In many cases, previously known factors were discovered and validated using genome-wide analyses. The set of protein sequences for which dynamical influence can be calculated is smaller and potentially biased. First, dynamical influence considers effects on network dynamics after stimulus, so it is not applicable to proteins that do not respond to any stimuli. Second, we calculated dynamical influence from mathematical models, and such models exist for only some systems. Lastly, we calculated dynamical influence at the domain level, so we did not consider the evolution of linker sequences between domains. Because the previously known factors we consider are defined at the whole-protein level, they can never fully explain evolutionary rate at the domain level. Nevertheless, we used correlation analysis to understand how dynamical influence compares with previously known predictors of evolutionary rate, for the set of networks and protein domains represented in our study.

In multicellular organisms, proteins that are expressed in more cell types (i.e., have higher expression breadth) evolve more slowly [65], and this is true in the vertebrate networks we study (Table 1). The significant positive correlation between dynamical influence and expression breadth (Table 1) suggests that protein domains with key roles in these networks exert their effects across multiple tissues, providing a functional explanation for the observed correlation between expression breadth and evolutionary rate.

Expanding from expression breadth, the strongest known correlate with protein evolutionary rate is expression level. Proteins with greater expression evolve more slowly in both yeast [66] and vertebrates [67], which may reflect the costs of protein mis-folding [15, 68–70] or mis-interaction [71]. In our networks, we found the expected negative correlation between evolutionary rate and expression level (Table 1). That estimated mean correlation is weaker than that between evolutionary rate and dynamical influence (Table 1), although the confidence intervals overlap. Indeed, dynamical influence is not significantly correlated with expression level (Table 1), indicating that dynamical influence reveals previously unanticipated evolutionary pressures beyond the strongest previously known correlate.

A significant advantage of our approach is that it captures how molecular inputs are integrated into functional phenotypic outcomes that may be selected upon. One aspect of network biology that has been previously considered in determining protein evolution is topology. Specifically, proteins with more interaction partners (i.e., greater degree) [72] or more central locations within networks (greater betweenness centrality) [73] evolve more slowly. Consistent with this previous work, we find that domain evolutionary rate has a significant negative correlation with both protein degree and betweenness centrality (Table 1). But, intriguingly, dynamical influence of protein domains is not significantly correlated with degree or betweenness centrality (Table 1) of the corresponding proteins. Why is the influence of topology not captured in our dynamics-based analysis of evolutionary rate? Network topology is a crude measure of function; networks with the same topology can have different dynamics and thus different functions [74]. Thus, our focus on network dynamics rather than topology provides novel insight into protein domain evolution by directly quantifying system output.

Expression level and network topology are also represented in the models themselves, by the total abundance of the molecular species that represent each protein and by the reactions that connect them. The correlations of dynamical influence and evolutionary rate with these model-derived quantities (S5 Table) are similar to those with experimentally-derived expression and topology (Table 1). In fact, the partial correlation between dynamical influence and evolutionary rate controlling for expression and topology is stronger when using model-derived values than when using experimental values. The relationship we find between dynamical influence and evolutionary rate is thus not driven by hidden co-variation between dynamical influence and abundance or topology within the models.

These assessments of dynamical influence relative to known contributors to protein evolution clearly indicate that our approach has uncovered previously unappreciated constraints on protein evolution. Is this new insight sufficient to explain the conundrums raised by knockout experiments? In our data, we found that the correlation between evolutionary rate and knockout measures of function was so weak as to be nonsignificant (Table 1), consistent with prior work [11, 12]. Strikingly, across the eleven vertebrate networks that include both essential and non-essential proteins and the six yeast networks (for which knockout growth rate data is available), we find no statistical correlation between dynamical influence and essentiality or knockout growth rate (Table 1). Thus, the highly significant correlation between dynamical influence and evolutionary rate (Fig 2, Table 1) provides a new perspective on the influence of protein function on evolutionary rate.

But, evolutionary rates are complex and likely integrate selection on multiple processes [2–5]. To assess the power of our approach in comparison with alternative integrative analyses, we used partial correlation analysis [12]. Across all our networks, we find that when expression, network topology, and knockout effect are controlled for, the mean correlation between protein domain evolutionary rate and dynamical influence remains statistically significant (Fig 3 and Table 1). Because the predictive power of dynamical influence cannot be explained by other factors, it provides novel and previously inaccessible insight into evolutionary rates within protein networks.

The existence of overlapping protein domains might inflate statistical significance in our analyses across models. To account for this, for all domains that appeared in more than one model, we randomly kept each domain in one of the models and deleted it from the others. We did this randomization one thousand times and repeated our correlation analysis each time, obtaining distributions of mean correlations and permutation p-values. There was a tail of large p-values for the fully controlled correlation between dynamical influence and evolutionary rate, corresponding to randomizations in which many domains happened to be retained in the few models with a positive correlation (S1 Fig). The median results we found were, however, similar to our analysis using all the data (S6 Table), suggesting that overlapping domains do not substantially affect our statistics.

Dynamical systems biology models offer great promise for developing and testing evolutionary hypotheses [21, 75]. Previous topological and flux-balance analysis of networks has offered insight into protein evolution [76–78], but dynamical models contribute substantial biological detail not previously captured by these approaches. We have shown that incorporating that detail can, for domains within dynamical networks, explain the previous lack of correlation between protein function and evolutionary rate. Dynamical models have previously been used to predict the phenotypic effects of mutations [79] and to assess the correlation between network sensitivity and protein evolution in phototransduction [80] and in pyrimidne biosynthesis [81]. Here we consider many networks to reveal a previously unexplored and general link between dynamical influence and protein domain evolutionary rate within networks. Given the rapid pace of progress in systems biology modeling [82], the anticipated advances in model scope and validation will provide even more robust data sets to uncover previously unanticipated factors influencing evolutionary processes.

## Materials and Methods

### Dynamical influence

We defined the dynamical influence *κ*_*i*_ of reaction rate constant *k*_*i*_ by

$$\kappa_{i}^{2}=\sum_{\text{stimulation conditions}\;c}\;\;\;\sum_{\text{molecular species}\;y}{\left.\frac{1}{T_{c}}\int_{0}^{T_{c}}{\left(\frac{dy_{c}\left(t\right)}{dk_{i}}\frac{k_{i}}{y_{\text{max}}}\right)}^{2}\right|}_{k=k^{*}}\;\;\;\;\;\;dt.$$ (1)

Here *y*_*c*_(*t*) is the time course of molecular species *y* in condition *c*, evaluated using the rate constant values k* from the original publication. The derivative *dy*_*c*_(*t*)/*dk*_*i*_ of the time course with respect to rate constant *k*_*i*_ measures how sensitive that molecular species or metabolite is to changes in that rate constant. To make relative comparisons, we normalized these sensitivities by the value *k*_*i*_ of the rate constant and the maximum *y*_max_ of molecular species *y* over all stimulation conditions. We normalized by *y*_max_ rather than using a control coefficient $\frac{dy(t)}{dk}\frac{k}{y(t)}=\frac{d\ln y(t)}{d\ln k}$ [83] because many molecular species in signaling models begin with zero concentration, so the control coefficient would be undefined. We found the total effect of changes in *k*_*i*_ by squaring these normalized sensitivities, integrating over the time course of each stimulation condition, and summing over all molecular species and stimulation conditions.

We defined the dynamical influence *D*_*d*_ of protein domain *d* to be the geometric mean of the influences *κ* of the *N*_*d*_ reaction rate constants for reactions in which that domain participates:

$$D_{d}={\left(\prod_{r=1}^{N_{d}}\kappa_{r}\right)}^{\frac{1}{N_{d}}}.$$ (2)

We took a geometric mean because rate constant sensitivities range over orders of magnitude [61].

Stochastic noise plays an important role in many cellular networks [84, 85]. In those cases, networks are not well-modeled by ordinary differential equations (ODEs), although parameter sensitivities can be defined and calculated [86]. To minimize the complications introduced by stochasticity, we focused on models of signaling and biosynthesis in which concentrations of molecular species were sufficiently large to justify a continuous approximation to probabilistic biochemical reaction rates [57]. Moreover, because sources and levels of gene expression noise are known to vary according to initial conditions [87], we restricted our analysis to models which were fit to experimental data arising from multiple initial conditions and measuring multiple reporters.

We downloaded systems biology models in Systems Biology Markup Language (SBML) format [88] from the Feb. 8, 2012 release of BioModels [25]. We calculated dynamical influence for all protein-related biological parameters in each model, using SloppyCell [89] and simulating under the conditions considered in each model’s original paper (S1 Text). These parameters represent a variety of biological phenomena, such as binding and catalytic constants and rates of diffusion and production. We considered only those parameters representing rates of biochemical reactions that depend on protein structure, because we expected constraint on those reactions to have the strongest effect on evolutionary rates. Given the dynamical influences *κ* for each reaction constant, we reviewed the literature to determine the protein domain or domains at which the reaction occurs, and we assigned those influences to that domain or domains (S1 Dataset).

### Evolutionary rates

UniProt protein ID’s were acquired from the BioModels annotation in the SBML file for each model and converted to NCBI Protein IDs for vertebrates or open reading frame (ORF) numbers for yeast. Some models specified more than one Uniprot ID for a single protein, in cases where there is more than one transcript identified and both appear to perform the same function (for example, MEK1 and MEK2). Where more than one Uniprot ID was specified, we reviewed the model publication and the protein network literature to select a single transcript. In the case of metabolic flux models that track metabolites rather than proteins, we used the names of the enzymes involved in the reactions to find the appropriate protein identifier.

Vertebrate homologous protein alignments were downloaded from the NCBI Homologene database [90], and for each protein in the alignment, nucleotide sequence was downloaded from NCBI Entrez [90]. These nucleotide sequences were then used as a template to back-translate the Homologene protein alignments to nucleotide alignments. Yeast gene information for the 7 species in the tree in S2 Fig was downloaded from the Saccharomyces Genome Database [91] on Nov. 19, 2012. These gene sequences were translated to protein amino acid sequences using Biopython [92], aligned using ClustalW [93], and then back-translated to aligned nucleotide sequence using the gene sequence as a template.

Protein domain annotation was done manually using literature review, based on information for the human protein in vertebrate models or the *Sa. cerevisiae* protein in yeast. Evolutionary rates were calculated using codeML from PAML Version 4.4b [94], with one dN/dS ratio per tree (model 0), the F3x4 codon substitution model, and a rooted tree, as in [95]. The Mgene = 3 setting of codeml was used to estimate a single dN/dS ratio per annotated protein domain. We required a minimum of 4 homologs to include a gene in the analysis, and for each gene any species with more than one homologue was excluded. Because instability is a concern when estimating multiple dN/dS ratios for a single protein sequence, we iterated each codeml run until we acquired three models for which the log-likelihood was within 0.01 of the lowest log-likelihood obtained and then used the model with the lowest log-likelihood.

### Gene expression and specificity

Vertebrate gene expression and tissue specificity data was compiled from the mouse GNF1M dataset [96], downloaded from http://bioGPS.org/downloads. The data consist of microarray probes for a number of tissue types, with each probe’s name including the corresponding gene name, which we mapped to Ensembl gene IDs using Ensembl BioMart [97]. We restricted our analysis to normal adult tissues as in Fig S2 of [95]. To calculate the expression level corresponding to each microarray probe, we took the arithmetic average over replicates of the same tissue and then took the geometric average over tissues. To calculate the expression level of each gene, we then took the arithmetic average of the probe expression levels corresponding to that gene.

Yeast expression data [98] was obtained from http://younglab.wi.mit.edu/pub/data/orf_transcriptome.txt and used without modification.

Protein abundance within each model was calculated as the sum of initial conditions for all molecular species corresponding to a given protein, including modified forms and complexes. None of the models we considered included transcription or translation, so total levels of all proteins were constant throughout the simulations.

### Gene essentiality and dispensibility

We downloaded mouse knockout phenotype data from the Mouse Genome Informatics database [99] at http://www.informatics.jax.org/phenotypes.shtml on July 11, 2011. We assembled phenotype information for homozygous knockouts and coded a gene as essential if it resulted in one of the following phenotypes: abnormal reproductive system physiology, prenatal lethality, perinatal lethality, postnatal lethality, premature death, abnormal reproductive system morphology, lethality at weaning, preweaning lethality, partial lethality, and all sub-phenotypes of these phenotypes. If homozygous knockout of a gene did not cause one or more of these phenotypes we coded it as non-essential. To validate our parsing of this data, we compared against the results of [12].

Data for yeast knockout growth rate on YPD media were obtained from the file Regression_Tc1_hom.txt downloaded from the Stanford YDPM database http://www-deletion.stanford.edu/YDPM/YDPM_index.html on March 13, 2013.

### Network degree and centrality

We downloaded protein-protein interaction data for both humans and yeast from the Interologous Interaction Database [100] on April 20, 2012. These data take the form of a list of interactions between two proteins, and the dataset from which the interaction was curated. Because we were interested in experimentally verified interactions we restricted our analysis to the HPRD, BIND, IntAct, and INNATEDB datasets for humans and the Krogan_Core, Yu_GoldStd, YeastHigh, YeastLow, and BIND datasets for yeast. We used the python package NetworkX [101] to load these lists of interactions and compute each protein’s degree and its betweenness centrality, which is the fraction of all of the shortest paths between protein pairs in the network that pass through that protein. Model-derived network degree and centrality were calculated from a graph with nodes for each domain in the model and edges between any pairs of domains that participate in a reaction together.

### Statistical analyses

Dynamical influence and evolutionary rate are defined at the domain level, but all other factors in Table 1 are defined at the protein level, so in our statistical analyses these other factors were assumed to be equal for all domains within a given protein. We used partial correlation analysis to assess the degree to which these factors account for the observed relationship between dynamical influence and domain evolutionary rate. To do so, within each model we fit linear models for dynamical influence and evolutionary rate as a function of all the other factors and then computed the product-moment correlation between the residuals from these models. Only domains for which all variables were measured were included in these partial correlations.

To analyze correlations and partial correlations across models (Table 1, Fig 3, and S5 Table), we applied the random-effects meta-analysis approach of Hunter and Schmidt [52]. In this approach, the mean correlation *ρ*_0_ in the population is estimated by the average of the observed correlations *r* of the sampled models, weighted by the sample size *n* of domains with relevant data in each model. So the estimated mean correlation is

$${\hat{\rho }}_{0}=\frac{\sum n_{i}r_{i}}{\sum n_{i}},$$ (3)

where sums here and below are over models (Eq. 3.1 in [52]). The variance across samples from the population $\sigma_{r}^{2}$ is the sum of the variance in population correlations $\sigma_{\rho }^{2}$ and the variance due to sampling error $\sigma_{e}^{2}$:

$$\sigma_{r}^{2}=\sigma_{\rho }^{2}+\sigma_{e}^{2}.$$ (4)

The variance across samples can be estimated as [52]

$${\hat{\sigma }}_{r}^{2}=\frac{\sum n_{i}{\left(r_{i}-{\hat{\rho }}_{0}\right)}^{2}}{\sum n_{i}}.$$ (5)

The sampling variance can be estimated as (Eq. 3.5 in [52])

$${\hat{\sigma }}_{e}^{2}=\frac{\sum \frac{n_{i}{\left(1-r_{i}^{2}\right)}^{2}}{n_{i}-1}}{\sum n_{i}}.$$ (6)

The standard deviation *σ*_*ρ*_ of the population correlations sets the width of the distribution curves in Fig 3, and it can be estimated by solving Eq 4 for *σ*_*ρ*_ and substituting in the estimates ${\hat{\sigma }}_{e}$ and ${\hat{\sigma }}_{r}$. The standard error $SE({\hat{\rho }}_{0})$ of the estimated mean correlation ${\hat{\rho }}_{0}$ depends on the number of population samples *K*, which is 18 here (Eq. 5.1 in [52]):

$$SE\left({\hat{\rho }}_{0}\right)={\hat{\sigma }}_{r}/\sqrt{K}.$$ (7)

The 95% confidence intervals reported in Table 1 and S5 Table are thus ${\hat{\rho }}_{0}\pm 1.96\;SE\left({\hat{\rho }}_{0}\right)$. The most popular alternative approach for random-effects meta-analysis of correlations, developed by Hedges and colleagues [102], estimates the population mean correlation *ρ*_0_ and the standard error of that estimate $SE({\hat{\rho }}_{0})$ using more complex weightings based on Fisher’s *r*-to-*Z* transform. We adopted the Hunter and Schmidt approach because simulation studies suggest that it produces more accurate estimates of the population mean correlation and more accurate confidence intervals when variation in the population is large [56].

In our permutation tests, our null model was that dynamical influence or evolutionary rate was uncorrelated with other protein domain properties (Table 1). To generate null distributions of correlations, we permuted dynamical influences, evolutionary rates, and all other factors within each model. Because domains share reactions, their influences are not independent, and thus we could not simply permute them to simulate our null model. Instead, we permuted the influences of reaction parameters, which are the most basic unit of our analysis, and we then recalculated the influence for each domain based on the new sets of parameter influences. Evolutionary rates are defined at the domain level, so we simply permuted them within each model. The other factors are defined at the protein level, and we permuted them at the protein level, so that domains within the same protein would still always, for example, have the same expression level. To generate the null distribution for the partial correlation between domain evolutionary rate and dynamical influence, controlling for other variables, we permuted the residuals from the linear models used to calculate the partial correlation [103, 104]. This approach disrupts any relationship between domain evolutionary rate and dynamical influence while preserving all other relationships between network variables. We permuted variables or residuals separately within each model and then used Eq 3 to calculate mean correlations across models for each permutation. After carrying out 10,000 permutations, the two-sided p-values we report (Table 1) are the quantiles of the real data absolute mean correlations among the permuted absolute mean correlations. In all cases, the permutation test results are compatible with the 95% confidence intervals (Table 1); smaller p-values correspond to confidence intervals that more strongly exclude zero. Note that permutation tests based on correlation or partial correlation coefficients are strictly tests of the null hypothesis that the two variables are independent [105]. Thus our permutation test cannot reject the possibility that the considered variables are dependent but uncorrelated.

## Supporting Information

S1 Text Details of analysis for each model. (PDF)

S1 Table Correlations in vertebrate models. Spearman rank (*ρ*) and rank biserial (*rb*) correlation coefficients for variables evolutionary rate dN/dS (*ω*), dynamical influence (*D*), expression breadth (*B*), expression level (*X*), interaction degree (*d*), interaction betweenness centrality (*C*), and knock-out essentiality (*E*). Domains with missing values for any correlate were dropped prior to calculating correlations, and *N* represents the number of domains used in the analysis. For correlations, p-values were calculated via permutation of the data as described in the main text. For partial correlations, p-values were calculated by permutation of the residuals from the linear models [103, 104].
(PDF)

S2 Table Correlations in yeast models. As in S1 Table, but for yeast models, which have knock-out growth rate (*Gr*) data.
(PDF)

S3 Table Between-model correlations between protein domain dynamical influences. For each pair of models with at least four overlapping domains, shown is the Spearman rank correlation and number of overlapping domains.
(PDF)

S4 TableDynamical influence calculated with finite versus differential perturbations.Dynamical influence was recalculated using finite perturbations $\frac{\Delta y}{\Delta k}=\frac{y\left(k^{+}\right)-y\left(k^{-}\right)}{k^{+}-k^{-}}$ instead of differential perturbations $\frac{dy}{dk}$ (Eq 1), with *k*^+^ and *k*^−^ being ±25% perturbations of each parameter. Tabulated are the rank correlations between domain influences calculated using finite and differential perturbations. The almost perfect correlations suggest that our dynamical influence analysis also applies to mutations of moderate effect.(PDF)

S5 Table Overall correlations calculated with model-derived expression and topology data. Meta-analysis confidence intervals and permutation p-values as in Table 1. Spearman rank (*ρ*) correlation coefficients for variables evolutionary rate dN/dS (*ω*), dynamical influence (*D*), model-derived expression (*MX*), model-derived interaction degree (*Md*), model-derived interaction centrality (*MC*), expression breadth (*B*), knock-out essentiality (*E*), and knock-out growth rate (*Gr*).
(PDF)

S6 Table Overall correlations with overlapping domains removed. Meta-analysis mean correlations and permutation p-values as in Table 1, but without overlapping domains between models. Values are the 50th (5th, 95th) quantiles of correlations and p-values calculated from 1000 runs in which each domain that appears in multiple models was considered for only a single randomly chosen model.
(PDF)

S1 Fig Distributions of overall correlation and p-value when overlapping domains are removed. Shown are the full distributions that are summarized in the last row of S6 Table. A: The partial correlation between domain dynamical influence and evolutionary rate is negative for all randomizations. B: The distribution of p-values is strongly concentrated below 0.05. The tail of larger p-values is generated by randomizations that concentrate domains in the few models with a positive correlation.
(PDF)

S2 Fig Phylogenetic trees for species used in this study. A: Vertebrates. B: Yeasts. In both, branch lengths represent amino acid divergence.
(PDF)

S1 Dataset Complete model and protein domain annotation, including covariate data for each domain. Each model corresponds to two sheets. The first sheet contains the reaction parameters, their dynamical influences, and the reactions they correspond to. The second sheet contains the protein and domain data, including assignment of reactions to domains and corresponding references (as PubMed IDs).
(XLSX)
