# Supplementary material for: Selection on Network Dynamics Drives Differential Rates of Protein Domain Evolution
Source: PLoS Genet. 2016 Jul 5;12(7):e1006132. doi: 10.1371/journal.pgen.1006132 (PMC4933380; doi:10.1371/journal.pgen.1006132)
Supplement: S1 Text — (PDF) [file pgen.1006132.s001.pdf]

Supporting Information

Brian K. Mannakee[1] and Ryan N. Gutenkunst*[2]

[1]Division of Epidemiology and Biostatistics,
Mel and Enid Zuckerman College of Public Health, University of Arizona
[2]Department of Molecular and Cellular Biology, University of Arizona

Models considered

1 EGF/NGF signaling [1]

Brown et al. developed a dynamic model of the EGF/NGF signaling network in rat pheochromocytoma (PC12) cells. We downloaded the SBML file `BIOMD0000000033.xml` from the BioModels database and used it without modification. We simulated this model under two different conditions, 100 ng/ml EGF and 50 ng/ml NGF, as described in [1]. Protein domain, parameter, and reaction assignments are in S1 Dataset. In addition to our primary analysis, we calculated dynamical influence over a restricted subset of proteins containing only activated Erk.

2 Arachodonic acid signaling [2]

In order to gain insights related to anti-inflammatory drug interaction and design, Yang et al. created and studied a dynamic model of the arachadonic acid signaling network in human polymorphonuclear leukocytes. We downloaded the SBML file `BIOMD0000000106.xml` from the BioModels database and used it without modification, integrating the model between 0 and 60 minutes as in [2]. The model contains reactions that create and destroy each tracked biomolecule. We excluded these because they are not reactions between modeled proteins. Protein domain, parameter, and reaction assignments are in S1 Dataset. In addition to our primary analysis, we calculated dynamical influence over a restricted subset of proteins containing ω-LTB4 and 15-Hete.

3 EGF/NGF signaling [3]

Sasagawa et al. developed a model of ERK signaling networks, with parameters derived by fitting model dynamics to *in vivo* dynamics in PC12 cells, and studied network dynamics under a variety of stimulation conditions. We downloaded SBML file `BIOMD0000000049.xml` from the BioModels database and used it without modification. For all conditions simulated we integrated the network from 0 to 3600 seconds (60 minutes) as in [3]. The SBML file is coded for constant stimulation by 10 ng/ml EGF, and this is the first of the conditions we simulated. We simulated constant stimulation by 10 ng/ml NGF by using SloppyCell to set EGF concentration to 0 and NGF concentration to 10 ng/ml. Sasagawa et al. also investigate the effect of ramping the concentration of EGF (or NGF) from 0 to 1.5 ng/ml over the course of the simulation. To accomplish this we created assignment rules in SloppyCell which updated the EGF (or NGF) concentration at each time step of the integration, setting it equal to 1.5 * (time/3600) ng/ml. As in the fixed simulation conditions, the network was stimulated by EGF or NGF, but not both. Protein domain, parameter, and reaction assignments are in S1 Dataset. In addition to our primary analysis, we calculated dynamical influence over a restricted subset of proteins containing only activated Erk.

*To whom correspondence should be addressed. Email: rgutenk@email.arizona.edu

4 EGF/MAPK cascade [4]

Schoeberl et al. modeled the EGF signaling pathway, comparing simulated time courses with experimental time courses in HeLa cells under several experimental conditions. We downloaded the SBML file `BIOMD0000000019.xml` from the BioModels database. The model specifies the value of parameter `k5` as a piecewise function of another parameter `C`, and piecewise functions are not supported by SloppyCell, so we removed the piecewise function from the SBML file and used SloppyCell to create two SBML events that replicate it. We simulated the model under three experimental conditions from [4], namely stimulus with 50, 0.5, and 0.25 ng/ml EGF. For all conditions we simulated from 0 to 60 minutes. The model includes receptor internalization reactions which is not modeled mechanistically, and we excluded these reactions from our analysis. Protein domain, parameter, and reaction assignments are in S1 Dataset. In addition to our primary analysis, we calculated dynamical influence over a restricted subset of proteins containing only activated Erk.

5 Myosin phosphorylation [5]

Maeda et al. developed a computational model of thrombin-dependent Rho-kinase activation and myosin light chain phosphorylation in human umbilical vein endothelial cells. We downloaded the SBML file `BIOMD0000000088.xml` for this model from the BioModels database. Some parameter names were duplicated, so we modified the model SBML file to assign unique parameter names and used the published parameter values (see S1 Dataset). The only simulation condition we considered was the $0.01\mu M$ thrombin stimulation published in the SBML file, and we integrated the model from 0 to 3600 seconds as in [5]. We ignored reactions occurring in the extra-cellular compartment, particularly thrombin/thrombin-receptor interactions, and assigned all other reactions to protein domains as outlined in S1 Dataset. We did not calculate a sensitivity for the parameter `ratio`, because it is always multiplied by another parameter `V_max` whose sensitivity we did calculate. SloppyCell interprets rate laws in terms of changes in concentration rather than changes in amount as called for by the SBML specification, so we adjusted reaction stoichiometries by a factor of 1/(compartment size) for reactants in compartment `c2`, the only compartment in all the models we consider with size not equal to 1. In addition to our primary analysis, we calculated dynamical influence over a restricted subset of proteins containing phosphorylated myosin light chain (pMLC) and phosphorylated myosin phosphatase targeting subunit 1 (pMYPT1).

6 Extrinsic apoptosis [6]

Albeck et al. developed a model of TRAIL-induced apoptosis and used it to analyze extrinsic apoptosis in HeLa cells. We downloaded the SBML file `BIOMD0000000220.xml` from the BioModels database and used it without modification. We simulated the model for 10 hours under the 50 ng/ml TRAIL stimulus condition encoded in the SBML file. We excluded extra-cellular reactions involving TRAIL, as well as intra-cellular reactions involving DISC and the TRAIL-DISC complex, because the protein components of the DISC are not specified in the model. We also excluded transport across the mitochondrial membrane and binding of proteins to the inner mitochondrial membrane, because these reactions are not mechanistically specified in the model. Protein domain, parameter, and reaction assignments are in S1 Dataset. In addition to our primary analysis, we calculated dynamical influence summing over a restricted subset of proteins containing Caspase 3, Caspase 8, cytosolic Smac, and cytosolic Cytochrome C.

7 EGF/Insulin crosstalk [7]

Borisov et al. developed a model of the Ras/Erk signaling system that incorporates mechanisms of cross-talk between the EGF and Insulin signaling pathways and tested it in HEK293 cells. We downloaded the SBML file `BIOMD0000000223.xml` from the BioModels database and used it without modification. This SBML model, however, was altered slightly from the originally published model by adding an extra-cellular compartment of size 34. While the model page on the BioModels website says this allows for the use of the original concentrations of EGF we found that this did not create the correct dynamics. Rather than altering the model by changing the size of the extra-cellular compartment we multiplied the desired concentrations of EGF by 34, which produced the correct model dynamics. We

simulated the model under four different experimental conditions, 0.01nM or 1 nM EGF with 0 or 100 nM Insulin, as described in [7]. This model contains a reaction in which Akt1 activates mTor via a chain reaction among a number of proteins that are not included in the model. As a result we only applied this reaction's sensitivity κ to the Akt1 kinase domain, but not to mTor. This is the only reaction in the model in which mTor appears, so mTor was excluded from our analysis of this model. Protein domain, parameter, and reaction assignments are in S1 Dataset. In addition to our primary analysis, we calculated dynamical influence summing over a restricted subset of proteins containing active (doubly phosphorylated) Erk and phosphorylated Akt.

8 G1 cell cycle progression [8]

Haberichter et al. constructed a dynamical model of mammalian G1 cell cycle progression in order to simulate cell cycle progression dynamics in proliferating cells continuously exposed to growth factors. We downloaded the SBML file `BIOMD0000000109.xml` from the BioModels database and used it without modification. We simulated the model under the experimental conditions encoded in the SBML file, integrating for 1000 minutes. The model includes reactions that create and destroy proteins, which we excluded from our analysis because they do not involve interaction with any other protein in the model. Protein domain, parameter, and reaction assignments are in S1 Dataset. In addition to our primary analysis, we calculated dynamical influence summing over a restricted subset of proteins containing the two activation states, hypo- and hyper-phosphorylated, of retinoblastoma tumor suppressor protein pRb.

9 ErbB signaling [9]

Birtwistle et al. built a model of ErbB signaling that describes the response of the signaling network to stimulus of all four ErbB receptors with EGF and HRG (heregulin), comparing model dynamics to dynamics in MCF-7 human breast cancer cells. We downloaded SBML file `BIOMD0000000175.xml` from the BioModels database and used it without modification. Experimental conditions in the paper include stimulation with 0 nM, 0.5 nM, and 10 nM EGF and HRG in each possible combination of those three stimuli, for a total of 12 experimental conditions, and we simulate each of these 12 conditions for 2000 seconds. As in other models we excluded receptor internalization reactions that are not mechanistically described in the model. Protein domain, parameter, and reaction assignments are in S1 Dataset. In addition to our primary analysis, we calculated dynamical influence summing over a restricted subset of proteins containing active (doubly phosphorylated) Erk and phosphorylated Akt. In particular, we used the normalized active Erk and Akt concentrations described in [9].

10 Wnt/Erk crosstalk [10]

Kim et al. created a model of the Erk and Wnt pathways to investigate the effect of a positive feedback loop resulting from crosstalk between Wnt and Erk signaling, and they compared model dynamics with experimental results in HEK293 cells. We downloaded the SBML file `BIOMD0000000149.xml` from the BioModels database and used it without modification. This model includes a protein `X` which is postulated to mediate the feedback between the two pathways; it is transcribed as a result Wnt signaling and activates B-raf in the Erk network. We did not include reactions for transcription and degradation of protein `X`, because they are not mechanistically specified. We did, however, include the reaction in which protein `X` activates B-raf, which occurs on the Ras-binding domain (RBD) of B-raf, because binding at the RBD is the mode of activation of B-raf, and the reaction is modeled in the same way as Ras activation of B-raf. We excluded creation and destruction of β-catenin as well as degradation of Axin, since they are not mechanistically described in the model. Protein domain, parameter, and reaction assignments are in S1 Dataset. In addition to our primary analysis, we calculated dynamical influence summing over a restricted subset of proteins containing active (doubly phosphorylated) Erk and the β-catenin/TCF complex.

11 Rod phototransduction [11]

Dell'Orco et al. developed a model of rod phototransduction specifically aimed at describing the mechanism of light adaptation in rod cells, verifying it by reproducing the results of several pre-

vious light adaptation response experiments in mouse rod cells. We downloaded the SBML file `BIOMD0000000326.xml` from the BioModels database and used it with minor modification. We removed two piecewise assignment rules, because SloppyCell does not handle piecewise rules, and replaced them with SBML events. We simulated six flash intensities, replicating those used to create Figure 7 in the publication, by setting the parameter `flash0Mag` to 1.54, 12.5, 45.8, 184, 800, and 2000. The parameter `kP1_rev` represents the rate of dissociation of phosphodiesterase from activated G-alpha molecules, and is set to zero in this model, so we excluded it from our analysis. Protein domain, parameter, and reaction assignments are in S1 Dataset. In addition to our primary analysis, we calculated dynamical influence summing over a restricted subset of molecular species containing only cyclic GMP.

12 IL-6 signaling [12]

Singh et al. developed a model of IL-6 signaling encompassing the Jak/STAT as well as MAP kinase pathways in human hepatocytes. We downloaded the SBML file `BIOMD0000000151.xml` from the BioModels database and used it without modification. We simulated the experimental conditions encoded in the model, 10 ng/ml IL-6 stimulus and an initial Shp2 concentration of 100 nM. The model includes transcription, translation, and mRNA translocation for the protein SOCS which are not mechanistically detailed, so we excluded them. Protein domain, parameter, and reaction assignments are in S1 Dataset. In addition to our primary analysis, we calculated dynamical influence summing over a restricted subset of proteins containing only active STAT3 dimers in the nucleus.

13 Trehalose biosynthesis [13]

Smallbone et al. created a model of the trehalose biosynthesis pathway in *Sa. cerevisiae.* We downloaded the SBML file `BIOMD0000000380.xml` from the BioModels database and used it without modification. This model reaches a steady state, and the model was validated by comparing the steady state concentrations of the metabolites in the model with experimental results in yeast experiencing heat shock. We simulated the model under the heat shock condition used in the publication, and ran the model for 50000 seconds, at which point all metabolites had reached steady state concentrations. The model creates `Clb2` in a reaction that is not mechanistically specified, and we excluded this reaction from our analysis. Protein domain, parameter, and reaction assignments are in S1 Dataset. In addition to our primary analysis, we calculated dynamical influence summing over only the metabolite of interest, trehalose-6-phosphate.

14 Glycolysis [14]

Talser et al. created a model of carbohydrate flux under oxidative stress conditions in *Sa. cerevisiae.* We downloaded the SBML file `BIOMD0000000247.xml` from the BioModels database and used it with substantial modification. The model file in the BioModels database included unfitted parameter values, but model author Markus Ralser generously provided us with the parameter values they obtained by fitting the model to experimental data, and we reparameterized the SBML file accordingly. We simulated the wild-type experimental conditions encoded in the model for 100 minutes. Protein domain, parameter, and reaction assignments are in S1 Dataset. In addition to our primary analysis, we calculated dynamical influence summing over the ratio of NADH to NADPH, the quantity of interest.

15 Cell cycle regulation [15]

Chen et al. developed a model of the cell cycle in *Sa. cerevisiae* in order to investigate the complex mechanisms of cell cycle control. We downloaded the SBML file `BIOMD0000000056.xml` from the BioModels database and used it with substantial modification. This model was constructed with some reactions combined into assignment rules, making it impossible to use SloppyCell to calculate dynamical influence for individual reaction parameters. We separated these assignment rules into individual reactions and added those reactions to the model file, replacing the assignment rules. In order to verify that the modified model was correct we replicated Figures 3 and 6 from the publication. The model contains reactions causing the degradation of various proteins by SCF, which is not included in the model, and these reactions are not mechanistically described, so we excluded them from our analysis. We simulated the wild-type experimental conditions encoded in the paper for 200 minutes

as in Figures 3 and 6 of the publication. Protein domain, parameter, and reaction assignments are in S1 Dataset. In addition to our primary analysis, we calculated dynamical influence summing over measures of the timing of cell cycle events; MASS, BUD, ORI, and SPN.

16 Mitotic exit [16]

Queralt et al. developed a model of the initiation of mitotic exit in *Sa. cerevisiae* induced by down-regulation of the phosphatase Cdc50. We downloaded the SBML file `BIOMD0000000409.xml` from the BioModels database and used it without modification. This model contains reactions creating and destroying proteins Clb2, Cdc20, securin, separase, Cdc5, and Cdc15 which are not mechanistically described in the model, and we excluded these reactions from our analysis. We simulated the wild type conditions encoded in the model for 50 minutes, as in Figure 7 of the publication. Protein domain, parameter, and reaction assignments are in S1 Dataset. In addition to our primary analysis, we calculated dynamical influence summing over only the concentration of active separase.

17 Mitotic exit [17]

Vinod et al. created a computational model of mitotic exit in *Sa. cerevisiae* aimed at investigating the role of separase and Cdc14 endocycles. We downloaded the SBML file `BIOMD0000000370.xml` from the BioModels database and used it with substantial modification. This model was constructed using rate rules rather than reactions in SBML. Because SloppyCell our analysis is focused on reactions, we converted the rate rules to reactions, ensuring that the model remained correct by using it to generate Figure 2 from the publication. The model includes reactions creating, destroying, or (de)activating proteins Clb2, Sic1, Cln1, Cdc20, Cdh1, Swi5, Pds1, Esp1, Cdc5, Polo, and MBF which are not mechanistically detailed, and we excluded these reactions from our analysis. We simulated the wild-type experimental conditions encoded in the model for 120 minutes as in Figure 2 of the publication. Protein domain, parameter, and reaction assignments are in S1 Dataset. In addition to our primary analysis, we calculated dynamical influence summing over only the concentration of active separase.

18 Pheromone pathway [18]

Kofahl and Klipp modelled the dynamics of the *Sa. cerevisiae* pheromone pathway. We downloaded the SBML file `BIOMD0000000037.xml` from the BioModels database and used it without modification. The model includes reactions for the destruction of `Ste2` and the export of `Bar` that are not mechanistically detailed, and we excluded them from our analysis. We simulated the wild-type experimental conditions encoded in the model for 30 minutes, as in [18]. Protein domain, parameter, and reaction assignments are in S1 Dataset. In addition to our primary analysis, we calculated dynamical influence summing over only the concentration of complexes M and N, which include Far1 and are required for polarized growth and cell cycle arrest respectively.

S1 Dataset

References are [19, 20, 21, 22, 23, 24, 25, 26, 27, 28, 29, 30, 31, 32, 33, 34, 35, 36, 37, 38, 39, 40, 41, 42, 43, 44, 45, 46, 47, 48, 49, 50, 51, 52, 53, 54, 55, 56, 57, 58, 59, 60, 61, 62, 63, 64, 65, 66, 67, 68, 69, 70, 71, 72, 73, 74, 75, 76, 77, 78, 79, 80, 81, 82, 83, 84, 85, 86, 87, 88, 89, 90, 91, 92, 93, 94, 95, 96, 97, 98, 99, 100, 101, 102, 103, 104, 105, 106, 107, 108, 109, 110, 111, 112, 113, 114, 115, 116, 117, 118, 119, 120, 121, 122, 123, 124, 125, 126, 127, 128, 129, 130, 16, 131, 132, 133, 134, 135, 136, 137, 138, 139, 140, 141, 142, 143, 144, 145, 146, 147, 148, 149, 150, 151, 152, 153, 154, 155, 156, 157, 158, 159, 160, 161, 162, 163, 164, 165, 166, 167, 168, 169, 170].

References

[1] Brown KS, Hill CC, Calero GA, Myers CR, Lee KH, Sethna JP, et al. The statistical mechanics of complex signaling networks: nerve growth factor signaling. Phys Biol. 2004 dec;1(3-4):184–95.

[2] Yang K, Ma W, Liang H, Ouyang Q, Tang C, Lai L. Dynamic simulations on the arachidonic acid metabolic network. PLoS Comput Biol. 2007 mar;3(3):e55.

[3] Sasagawa S, Ozaki YI, Fujita K, Kuroda S. Prediction and validation of the distinct dynamics of transient and sustained ERK activation. Nat Cell Biol. 2005 apr;7(4):365–73.

[4] Schoeberl B, Eichler-Jonsson C, Gilles ED, Müller G. Computational modeling of the dynamics of the MAP kinase cascade activated by surface and internalized EGF receptors. Nat Biotechnol. 2002 apr;20(4):370–5.

[5] Maeda A, Ozaki Yi, Sivakumaran S, Akiyama T, Urakubo H, Usami A, et al. Ca2+-independent phospholipase A2-dependent sustained Rho-kinase activation exhibits all-or-none response. Genes Cells. 2006 sep;11(9):1071–83.

[6] Albeck JG, Burke JM, Aldridge BB, Zhang M, Lauffenburger DA, Sorger PK. Quantitative analysis of pathways controlling extrinsic apoptosis in single cells. Mol Cell. 2008 apr;30(1):11–25.

[7] Borisov N, Aksamitiene E, Kiyatkin A, Legewie S, Berkhout J, Maiwald T, et al. Systems-level interactions between insulin-EGF networks amplify mitogenic signaling. Mol Syst Biol. 2009 jan;5(256):256.

[8] Haberichter T, Mädge B, Christopher Ra, Yoshioka N, Dhiman A, Miller R, et al. A systems biology dynamical model of mammalian G1 cell cycle progression. Mol Syst Biol. 2007 jan;3(84):84.

[9] Birtwistle MR, Hatakeyama M, Yumoto N, Ogunnaike BA, Hoek JB, Kholodenko BN. Ligand-dependent responses of the ErbB signaling network: experimental and modeling analyses. Mol Syst Biol. 2007 jan;3:144.

[10] Kim D, Rath O, Kolch W, Cho KH. A hidden oncogenic positive feedback loop caused by crosstalk between Wnt and ERK pathways. Oncogene. 2007 jul;26(31):4571–9.

[11] Dell'Orco D, Schmidt H, Mariani S, Fanelli F. Network-level analysis of light adaptation in rod cells under normal and altered conditions. Mol Biosyst. 2009 oct;5(10):1232–46.

[12] Singh A, Jayaraman A, Hahn J. Modeling regulatory mechanisms in IL-6 signal transduction in hepatocytes. Biotechnol Progr. 2006 dec;95(979):850–862.

[13] Smallbone K, Malys N, Messiha HL, Wishart JA, Simeonidis E. Building a kinetic model of trehalose biosynthesis in Saccharomyces cerevisiae. Methods Enzym. 2011 jan;500(null):355–70.

[14] Ralser M, Wamelink MM, Kowald A, Gerisch B, Heeren G, Struys Ea, et al. Dynamic rerouting of the carbohydrate flux is key to counteracting oxidative stress. J Biol. 2007 jan;6(4):10.

[15] Chen KCKC, Calzone L, Csikasz-Nagy A, Cross FR, Novak B, Tyson JJ. Integrative analysis of cell cycle control in budding yeast. Mol Biol Cell. 2004 aug;15(8):3841.

[16] Queralt E, Lehane C, Novak B, Uhlmann F. Downregulation of PP2A(Cdc55) phosphatase by separase initiates mitotic exit in budding yeast. Cell. 2006 may;125(4):719–32.

[17] Vinod PK, Freire P, Rattani A, Ciliberto A, Uhlmann F, Novak B. Computational modelling of mitotic exit in budding yeast: the role of separase and Cdc14 endocycles. J R Soc Interface. 2011 aug;8(61):1128–41.

[18] Kofahl B, Klipp E. Modelling the dynamics of the yeast pheromone pathway. Yeast. 2004 jul;21(10):831–50.

[19] Acehan D, Jiang X, Morgan DG, Heuser JE, Wang X, Akey CW, et al. Three-Dimensional Structure of the Apoptosome : Implications for Assembly , Procaspase-9 Binding , and Activation Southwestern Medical Center at Dallas. Structure. 2002;9:423–432.

[20] Adams JM, Cory S. The Bcl-2 apoptotic switch in cancer development and therapy. Oncogene. 2007 mar;26(9):1324–37.

[21] Adams MN, Ramachandran R, Yau MK, Suen JY, Fairlie DP, Hollenberg MD, et al. Structure, function and pathophysiology of protease activated receptors. Pharmacol Ther. 2011 jun;130(3):248–82.

[22] Apanovitch DM, Slep KC, Sigler PB, Dohlman HG. Sst2 is a GTPase-activating protein for Gpa1: purification and characterization of a cognate RGS-Galpha protein pair in yeast. Biochemistry. 1998 apr;37(14):4815–22.

[23] Asakawa K, Yoshida S, Otake F, Toh-e A. A novel functional domain of Cdc15 kinase is required for its interaction with Tem1 GTPase in Saccharomyces cerevisiae. Genetics. 2001 apr;157(4):1437–50.

[24] Azzam R, Chen SL, Shou W, Mah AS, Alexandru G, Nasmyth K, et al. Phosphorylation by cyclin B-Cdk underlies release of mitotic exit activator Cdc14 from the nucleolus. Science. 2004 jul;305(5683):516–9.

[25] Barbacci E, Guarino B, Stroh J. The Structural Basis for the Specificity of Epidermal Growth Factor and Heregulin Binding. J Biol Chem. 1995;.

[26] Barberis M, De Gioia L, Ruzzene M, Sarno S, Coccetti P, Fantucci P, et al. The yeast cyclin-dependent kinase inhibitor Sic1 and mammalian p27Kip1 are functional homologues with a structurally conserved inhibitory domain. Biochem J. 2005 may;387(Pt 3):639–47.

[27] Batzer AG, Rotin D, Ureña JM, Skolnik EY, Schlessinger J. Hierarchy of binding sites for Grb2 and Shc on the epidermal growth factor receptor. Mol Cell Biol. 1994 aug;14(8):5192–201.

[28] Bayascas JR. PDK1: the major transducer of PI 3-kinase actions. Curr Top Microbiol Immunol. 2010 jan;346:9–29.

[29] Bhunia A, Mohanram H, Bhattacharjya S. Structural determinants of the specificity of a membrane binding domain of the scaffold protein Ste5 of budding yeast: implications in signaling by the scaffold protein in MAPK pathway. Biochim Biophys Acta. 2012 may;1818(5):1250–60.

[30] Blain SW. Switching Cyclin D-Cdk4 kinase activity on and off. Cell Cycle. 2008;7(7):892–898.

[31] Bogner C, Leber B, Andrews DW. Apoptosis: embedded in membranes. Curr Opin Cell Biol. 2010 aug;22(6):845–851.

[32] Bos JL, Rehmann H, Wittinghofer A. GEFs and GAPs: critical elements in the control of small G proteins. Cell. 2007 jun;129(5):865–77.

[33] Boulares AH, Yakovlev AG, Ivanova V, Stoica BA, Wang G, Iyer S, et al. Role of poly(ADP-ribose) polymerase (PARP) cleavage in apoptosis. Caspase 3-resistant PARP mutant increases rates of apoptosis in transfected cells. J Biol Chem. 1999 aug;274(33):22932–40.

[34] Brown MT, Cooper JA. Regulation, substrates and functions of src. Biochim Biophys Acta. 1996 jun;1287(2-3):121–49.

[35] Burke JR, Deshong AJ, Pelton JG, Rubin SM. Phosphorylation-induced Conformational Changes in the Retinoblastoma Protein Inhibit E2F Transactivation Domain Binding * . J Biol Chem. 2010;285(21):16286–16293.

[36] Butty AC, Pryciak PM, Huang LS, Herskowitz I, Peter M. The role of Far1p in linking the heterotrimeric G protein to polarity establishment proteins during yeast mating. Science. 1998 nov;282(5393):1511–6.

[37] Calzada A, Sacristán M, Sánchez E, Bueno A. Cdc6 cooperates with Sic1 and Hct1 to inactivate mitotic cyclin-dependent kinases. Nature. 2001 jul;412(6844):355–8.

[38] Cameron AJM, Parker PJ. Advances in Enzyme Regulation Protein kinase C A family of protein kinases , allosteric effectors or both ? Adv Enzym Regul. 2010;50(1):169–177.

[39] Camps M. Catalytic Activation of the Phosphatase MKP-3 by ERK2 Mitogen-Activated Protein Kinase. Science. 1998 may;280(5367):1262–1265.

[40] Cheever ML, Snyder JT, Gershburg S, Siderovski DP, Harden TK, Sondek J. Crystal structure of the multifunctional Gbeta5-RGS9 complex. Nat Struct Mol Biol. 2008 feb;15(2):155–62.

[41] Chen X, Vinkemeier U, Zhao Y, Jeruzalmi D, Darnell JE, Kuriyan J. Crystal structure of a tyrosine phosphorylated STAT-1 dimer bound to DNA. Cell. 1998 may;93(5):827–39.

[42] Chen Y, Rai R, Zhou ZR, Kanoh J, Ribeyre C, Yang Y, et al. A conserved motif within RAP1 has diversified roles in telomere protection and regulation in different organisms. Nat Struct Mol Biol. 2011 feb;18(2):213–21.

[43] Chipuk JE, Moldoveanu T, Llambi F, Parsons MJ, Green DR. The BCL-2 family reunion. Mol Cell. 2010 feb;37(3):299–310.

[44] Cho JH, Muralidharan V, Vila-Perello M, Raleigh DP, Muir TW, Palmer AG. Tuning protein autoinhibition by domain destabilization. Nat Struct Mol Biol. 2011 may;18(5):550–5.

[45] Choi Y, Konopka JB. Accessibility of cysteine residues substituted into the cytoplasmic regions of the alpha-factor receptor identifies the intracellular residues that are available for G protein interaction. Biochemistry. 2006 dec;45(51):15310–7.

[46] Cote RH. Characteristics of photoreceptor PDE (PDE6): similarities and differences to PDE5. Int J Impot Res. 2004 jun;16 Suppl 1:S28–33.

[47] Coughlin SR. Thrombin signalling and protease-activated receptors. Nature. 2000 sep;407(6801):258–64.

[48] Dance M, Montagner A, Salles JP, Yart A, Raynal P. The molecular functions of Shp2 in the Ras/Mitogen-activated protein kinase (ERK1/2) pathway. Cell Signal. 2008 mar;20(3):453–9.

[49] Daran JM, Dallies N, Thines-Sempoux D, Paquet V, François J. Genetic and biochemical characterization of the UGP1 gene encoding the UDP-glucose pyrophosphorylase from Saccharomyces cerevisiae. Eur J Biochem. 1995 oct;233(2):520–30.

[50] De Virgilio C, Bürckert N, Bell W, Jenö P, Boller T, Wiemken A. Disruption of TPS2, the gene encoding the 100-kDa subunit of the trehalose-6-phosphate synthase/phosphatase complex in Saccharomyces cerevisiae, causes accumulation of trehalose-6-phosphate and loss of trehalose-6-phosphate phosphatase activity. Eur J Biochem. 1993 mar;212(2):315–23.

[51] Delgado ML, O'Connor JE, Azorín I, Renau-Piqueras J, Gil ML, Gozalbo D. The glyceraldehyde-3-phosphate dehydrogenase polypeptides encoded by the Saccharomyces cerevisiae TDH1, TDH2 and TDH3 genes are also cell wall proteins. Microbiology. 2001 feb;147(Pt 2):411–7.

[52] Dolan JW, Kirkman C, Fields S. The yeast STE12 protein binds to the DNA sequence mediating pheromone induction. Proc Natl Acad Sci U S A. 1989 aug;86(15):5703–7.

[53] Dowell SJ, Bishop AL, Dyos SL, Brown AJ, Whiteway MS. Mapping of a yeast G protein betagamma signaling interaction. Genetics. 1998 dec;150(4):1407–17.

[54] Dube P, Herzog F, Gieffers C, Sander B, Engel A, Riedel D, et al. Localization of the Coactivator Cdh1 and the Cullin Subunit Apc2 in a Cryo-Electron Microscopy Model of Vertebrate APC / C. Mol Cell. 2005;20:867–879.

[55] Fallon JL, Halling DB, Hamilton SL, Quiocho FA. Structure of calmodulin bound to the hydrophobic IQ domain of the cardiac Ca(v)1.2 calcium channel. Structure. 2005 dec;13(12):1881–6.

[56] Ferguson KM, Lemmon MA, Schlessinger J, Sigler PB. Structure of the high affinity complex of inositol trisphosphate with a phospholipase C pleckstrin homology domain. Cell. 1995 dec;83(6):1037–46.

[57] Ferrezuelo F, Aldea M, Futcher B. Bck2 is a phase-independent activator of cell cycle-regulated genes in yeast. Cell Cycle. 2009 jan;8(2):239–52.

[58] Gao C, Chen YG. Dishevelled : The hub of Wnt signaling. Cell Signal. 2010;22(5):717–727.

[59] Garrison TR, Zhang Y, Pausch M, Apanovitch D, Aebersold R, Dohlman HG. Feedback phosphorylation of an RGS protein by MAP kinase in yeast. J Biol Chem. 1999 dec;274(51):36387–91.

[60] Gartner A, Jovanović A, Jeoung DI, Bourlat S, Cross FR, Ammerer G. Pheromone-dependent G1 cell cycle arrest requires Far1 phosphorylation, but may not involve inhibition of Cdc28-Cln2 kinase, in vivo. Mol Cell Biol. 1998 jul;18(7):3681–91.

[61] George R, Schuller AC, Harris R, Ladbury JE. A phosphorylation-dependent gating mechanism controls the SH2 domain interactions of the Shc adaptor protein. J Mol Biol. 2008 mar;377(3):740–7.

[62] Geymonat M, Spanos A, de Bettignies G, Sedgwick SG. Lte1 contributes to Bfa1 localization rather than stimulating nucleotide exchange by Tem1. J Cell Biol. 2009 nov;187(4):497–511.

[63] Giorgione JR, Lin JH, McCammon JA, Newton AC. Increased Membrane Affinity of the C1 Domain of Protein Kinase C Compensates for the Lack of Involvement of Its C2 Domain in Membrane Recruitment *. J Biol Chem. 2006;281(3):1660–1669.

[64] Goh PY, Lim HH, Surana U. Cdc20 protein contains a destruction-box but, unlike Clb2, its proteolysisis not acutely dependent on the activity of anaphase-promoting complex. Eur J Biochem. 2000 jan;267(2):434–49.

[65] Golsteyn RM. Cdk1 and Cdk2 complexes (cyclin dependent kinases) in apoptosis : a role beyond the cell cycle. Cancer Lett. 2005;217:129–138.

[66] Good M, Tang G, Singleton J, Reményi A, Lim WA. The Ste5 scaffold directs mating signaling by catalytically unlocking the Fus3 MAP kinase for activation. Cell. 2009 mar;136(6):1085–97.

[67] Goto JI, Mikoshiba K. Inositol 1,4,5-Trisphosphate Receptor-Mediated Calcium Release in Purkinje Cells: From Molecular Mechanism to Behavior. Cerebellum. 2011 jun;.

[68] Gotoh N. Regulation of growth factor signaling by FRS2 family docking/scaffold adaptor proteins. Cancer Sci. 2008 jul;99(7):1319–25.

[69] Gottardi CJ, Gumbiner BM. Adhesion signaling : How β -catenin interacts with its partners. J Biol Chem. 2001;p. 792–794.

[70] Granovsky AE, Rosner MR. Raf kinase inhibitory protein : a signal transduction modulator and metastasis suppressor. Cell Res. 2008;18(4):452–457.

[71] Grassie ME, Moffat LD, Walsh MP, Macdonald JA. The myosin phosphatase targeting protein (MYPT) family: A regulated mechanism for achieving substrate specificity of the catalytic subunit of protein phosphatase type 1δ. Arch Biochem Biophys. 2011 jun;510(2):147–59.

[72] Grauslund M, R\onnow B. Carbon source-dependent transcriptional regulation of the mitochondrial glycerol-3-phosphate dehydrogenase gene, GUT2, from Saccharomyces cerevisiae. Can J Microbiol. 2000 dec;46(12):1096–100.

[73] Gray CH, Good VM, Tonks NK, Barford D. The structure of the cell cycle protein Cdc14 reveals a proline-directed protein phosphatase. EMBO J. 2003 jul;22(14):3524–35.

[74] Griffin JL, Bowler MW, Baxter NJ, Leigh KN, Dannatt HRW, Hounslow AM, et al. Near attack conformers dominate β-phosphoglucomutase complexes where geometry and charge distribution reflect those of substrate. Proc Natl Acad Sci U S A. 2012 may;109(18):6910–5.

[75] Guan KL, Figueroa C, Brtva TR, Zhu T, Taylor J, Barber TD, et al. Negative regulation of the serine/threonine kinase B-Raf by Akt. J Biol Chem. 2000 sep;275(35):27354–9.

[76] Gurevich VV, Hanson SM, Song X, Vishnivetskiy SA, Gurevich EV. The functional cycle of visual arrestins in photoreceptor cells. Prog Retin Eye Res. 2011 nov;30(6):405–30.

[77] Hanke S, Mann M. The Phosphotyrosine Interactome of the Insulin Receptor Family and Its Substrates. Mol Cell Proteomics. 2009;p. 519–534.

[78] Hendrickson C, Meyn MA, Morabito L, Holloway SL. The KEN box regulates Clb2 proteolysis in G1 and at the metaphase-to-anaphase transition. Curr Biol. 2001 nov;11(22):1781–7.

[79] Hilioti Z, Chung YS, Mochizuki Y, Hardy CF, Cohen-Fix O. The anaphase inhibitor Pds1 binds to the APC/C-associated protein Cdc20 in a destruction box-dependent manner. Curr Biol. 2001 sep;11(17):1347–52.

[80] Hong F, Haldeman BD, Jackson D, Carter M, Baker JE, Cremo CR. Biochemistry of smooth muscle myosin light chain kinase. Arch Biochem Biophys. 2011;510(2):135–146.

[81] Hornig NCD, Knowles PP, McDonald NQ, Uhlmann F. The dual mechanism of separase regulation by securin. Curr Biol. 2002 jun;12(12):973–82.

[82] Huang KN, Odinsky SA, Cross FR. Structure-function analysis of the Saccharomyces cerevisiae G1 cyclin Cln2. Mol Cell Biol. 1997 aug;17(8):4654–66.

[83] Huang Y, Rich RL, Myszka DG, Wu H. Requirement of both the second and third BIR domains for the relief of X-linked inhibitor of apoptosis protein (XIAP)-mediated caspase inhibition by Smac. J Biol Chem. 2003 dec;278(49):49517–22.

[84] Hubbard SR, Till JH. Protein tyrosine kinase structure and function. Annu Rev Biochem. 2000 jan;69:373–98.

[85] Hwang LH, Lau LF, Smith DL, Mistrot CA, Hardwick KG, Hwang ES, et al. Budding yeast Cdc20: a target of the spindle checkpoint. Science. 1998 feb;279(5353):1041–4.

[86] Ichiba T, Hashimoto Y, Nakaya M, Kuraishi Y, Tanaka S, Kurata T, et al. Activation of C3G guanine nucleotide exchange factor for Rap1 by phosphorylation of tyrosine 504. J Biol Chem. 1999 may;274(20):14376–81.

[87] Iyer VR, Horak CE, Scafe CS, Botstein D, Snyder M, Brown PO. Genomic binding sites of the yeast cell-cycle transcription factors SBF and MBF. Nature. 2001 jan;409(6819):533–8.

[88] Jaspersen SL, Charles JF, Morgan DO. Inhibitory phosphorylation of the APC regulator Hct1 is controlled by the kinase Cdc28 and the phosphatase Cdc14. Curr Biol. 1999 mar;9(5):227–36.

[89] Kaburagi Y, Momomura K, Yamamoto-Honda R, Tobe K, Tamori Y, Sakura H, et al. Site-directed mutagenesis of the juxtamembrane domain of the human insulin receptor. J Biol Chem. 1993 aug;268(22):16610–22.

[90] Kao S, Jaiswal RK, Kolch W, Landreth GE. Identification of the mechanisms regulating the differential activation of the mapk cascade by epidermal growth factor and nerve growth factor in PC12 cells. J Biol Chem. 2001 may;276(21):18169–77.

[91] Kast D, Espinoza-Fonseca LM, Yi C, Thomas DD. Phosphorylation-induced structural changes in smooth muscle myosin regulatory light chain. Proc Natl Acad Sci U S A. 2010 may;107(18):8207–12.

[92] Kiefer JR, Pawlitz JL, Moreland KT, Stegeman RA, Hood WF, Gierse JK, et al. Structural insights into the stereochemistry of the cyclooxygenase reaction. Nature. 2000 may;405(6782):97–101.

[93] Kikuchi A. Roles of Axin in the Wnt Signalling Pathway. Science. 1999;11(11):777–788.

[94] Kikuta Y, Kusunose E, Sumimoto H, Mizukami Y, Takeshige K, Sakaki T, et al. Purification and Characterization of Recombinant Human Neutrophil Leukotriene B 4 -Hydroxylase. Arch Biochem Biophys. 1998;355(2):201–205.

[95] Kobashigawa Y, Sakai M, Naito M, Yokochi M, Kumeta H, Makino Y, et al. Structural basis for the transforming activity of human cancer-related signaling adaptor protein CRK. Nat Struct Mol Biol. 2007 jun;14(6):503–10.

[96] Komolov KE, Senin II, Kovaleva Na, Christoph MP, Churumova Va, Grigoriev II, et al. Mechanism of rhodopsin kinase regulation by recoverin. J Neurochem. 2009 jul;110(1):72–9.

[97] Kraft C, Vodermaier HC, Maurer-Stroh S, Eisenhaber F, Peters JM. The WD40 propeller domain of Cdh1 functions as a destruction box receptor for APC/C substrates. Mol Cell. 2005 may;18(5):543–53.

[98] Kuhn H, Walther M, Kuban RJ. Mammalian arachidonate 15-lipoxygenases Structure , function , and biological implications. Prostaglandins Other Lipid Mediat. 2002;69:263–290.

[99] Lee KS, Park JE, Asano S, Park CJ. Yeast polo-like kinases: functionally conserved multitask mitotic regulators. Oncogene. 2005 jan;24(2):217–29.

[100] Leeuw T, Wu C, Schrag JD, Whiteway M, Thomas DY, Leberer E. Interaction of a G-protein beta-subunit with a conserved sequence in Ste20/PAK family protein kinases. Nature. 1998 jan;391(6663):191–5.

[101] Lehmann U, Sommer U, Smyczek T, Hörtner M, Frisch W, Volkmer-Engert R, et al. Determinants governing the potency of STAT3 activation via the individual STAT3-recruiting motifs of gp130. Cell Signal. 2006 jan;18(1):40–9.

[102] Lemmon MA. Ligand-induced ErbB receptor dimerization. Exp Cell Res. 2009 feb;315(4):638–48.

[103] Li H, Zhu H, Xu CJ, Yuan J. Cleavage of BID by caspase 8 mediates the mitochondrial damage in the Fas pathway of apoptosis. Cell. 1998 aug;94(4):491–501.

[104] Liu F, Hill DE, Chernoff J. Direct binding of the proline-rich region of protein tyrosine phosphatase 1B to the Src homology 3 domain of p130(Cas). J Biol Chem. 1996 dec;271(49):31290–5.

[105] Liu HY, Meakin SO. ShcB and ShcC activation by the Trk family of receptor tyrosine kinases. J Biol Chem. 2002 jul;277(29):26046–56.

[106] Lou M, Garrett TPJ, McKern NM, Hoyne Pa, Epa VC, Bentley JD, et al. The first three domains of the insulin receptor differ structurally from the insulin-like growth factor 1 receptor in the regions governing ligand specificity. Proc Natl Acad Sci U S A. 2006 aug;103(33):12429–34.

[107] MacKay VL, Welch SK, Insley MY, Manney TR, Holly J, Saari GC, et al. The Saccharomyces cerevisiae BAR1 gene encodes an exported protein with homology to pepsin. Proc Natl Acad Sci U S A. 1988 jan;85(1):55–9.

[108] Mashima R, Hishida Y, Tezuka T, Yamanashi Y. The roles of Dok family adapters in immunoreceptor signaling. Immunol Rev. 2009 nov;232(1):273–85.

[109] McBride HJ, Yu Y, Stillman DJ. Distinct regions of the Swi5 and Ace2 transcription factors are required for specific gene activation. J Biol Chem. 1999 jul;274(30):21029–36.

[110] McDonald CB, Seldeen KL, Deegan BJ, Farooq A. SH3 domains of Grb2 adaptor bind to PXpsiPXR motifs within the Sos1 nucleotide exchange factor in a discriminate manner. Biochemistry. 2009 may;48(19):4074–85.

[111] Mead J, Bruning AR, Gill MK, Steiner AM, Acton TB, Vershon AK. Interactions of the Mcm1 MADS box protein with cofactors that regulate mating in yeast. Mol Cell Biol. 2002 jul;22(13):4607–21.

[112] Meyn MA, Melloy PG, Li J, Holloway SL. The destruction box of the cyclin Clb2 binds the anaphase-promoting complex/cyclosome subunit Cdc23. Arch Biochem Biophys. 2002 nov;407(2):189–95.

[113] Miller ME, Cross FR, Groeger AL, Jameson KL. Identification of novel and conserved functional and structural elements of the G1 cyclin Cln3 important for interactions with the CDK Cdc28 in Saccharomyces cerevisiae. Yeast. 2005 oct;22(13):1021–36.

[114] Miller JJ, Summers MK, Hansen DV, Nachury MV, Lehman NL, Loktev A, et al. Emi1 stably binds and inhibits the anaphase-promoting complex / cyclosome as a pseudosubstrate inhibitor. Genes Dev. 2006;p. 2410–2420.

[115] Miller ML, Jensen LJ, Diella F, Jørgensen C, Tinti M, Li L, et al. Linear motif atlas for phosphorylation-dependent signaling. Sci Signal. 2008 jan;1(35):ra2.

[116] Mittag T, Marsh J, Grishaev A, Orlicky S, Lin H, Sicheri F, et al. Structure/function implications in a dynamic complex of the intrinsically disordered Sic1 with the Cdc4 subunit of an SCF ubiquitin ligase. Structure. 2010 mar;18(4):494–506.

[117] Mori S, Iwaoka R, Eto M, Ohki SY. Solution structure of the inhibitory phosphorylation domain of myosin phosphatase targeting subunit 1. Proteins. 2009 nov;77(3):732–5.

[118] Nagao K, Yanagida M. Securin can have a separase cleavage site by substitution mutations in the domain required for stabilization and inhibition of separase. Genes Cells. 2006 mar;11(3):247–60.

[119] Nicholson DW. Caspase structure, proteolytic substrates, and function during apoptotic cell death. Cell Death Differ. 1999 nov;6(11):1028–42.

[120] Nicholson SE, De Souza D, Fabri LJ, Corbin J, Willson TA, Zhang JG, et al. Suppressor of cytokine signaling-3 preferentially binds to the SHP-2-binding site on the shared cytokine receptor subunit gp130. Proc Natl Acad Sci U S A. 2000 jun;97(12):6493–8.

[121] Oberst A, Pop C, Tremblay AG, Blais V, Denault JB, Salvesen GS, et al. Inducible dimerization and inducible cleavage reveal a requirement for both processes in caspase-8 activation. J Biol Chem. 2010 mar;285(22):16632–16642.

[122] Oldham WM, Hamm HE. Heterotrimeric G protein activation by G-protein-coupled receptors. Nat Rev Mol Cell Biol. 2008 jan;9(1):60–71.

[123] Ory S, Zhou M, Conrads TP, Veenstra TD, Morrison DK. Protein phosphatase 2A positively regulates Ras signaling by dephosphorylating KSR1 and Raf-1 on critical 14-3-3 binding sites. Curr Biol. 2003 aug;13(16):1356–64.

[124] Ow YLP, Green DR, Hao Z, Mak TW. Cytochrome c: functions beyond respiration. Nat Rev Mol Cell Biol. 2008 jul;9(7):532–42.

[125] Pacold ME, Suire S, Perisic O, Lara-Gonzalez S, Davis CT, Walker EH, et al. Crystal structure and functional analysis of Ras binding to its effector phosphoinositide 3-kinase gamma. Cell. 2000 dec;103(6):931–43.

[126] Pearce LR, Komander D, Alessi DR. The nuts and bolts of AGC protein kinases. Nat Rev Mol Cell Biol. 2010 jan;11(1):9–22.

[127] Pearl LH, Barford D. Regulation of protein kinases in insulin, growth factor and Wnt signalling. Curr Opin Struct Biol. 2002 dec;12(6):761–7.

[128] Peter M, Gartner A, Horecka J, Ammerer G, Herskowitz I. FAR1 links the signal transduction pathway to the cell cycle machinery in yeast. Cell. 1993 may;73(4):747–60.

[129] Pinheiro AS, Marsh JA, Forman-Kay JD, Peti W. Structural signature of the MYPT1-PP1 interaction. J Am Chem Soc. 2011 jan;133(1):73–80.

[130] Queralt E, Igual JC. Functional distinction between Cln1p and Cln2p cyclins in the control of the Saccharomyces cerevisiae mitotic cycle. Genetics. 2004 sep;168(1):129–40.

[131] Raaijmakers JH, Bos JL. Specificity in Ras and Rap signaling. J Biol Chem. 2009 apr;284(17):10995–9.

[132] Roskoski R. RAF protein-serine/threonine kinases: structure and regulation. Biochem Biophys Res Commun. 2010 aug;399(3):313–7.

[133] Rudberg PC, Tholander F, Andberg M, Thunnissen MMGM, Haeggstro JZ. Leukotriene A 4 Hydrolase: Identification of a Common Carboxylate Recognition Site for the Epoxide Hydrolase and Aminopeptidase Substrates. Biochemistry. 2004;279(26):27376–27382.

[134] Savaskan NE, Ufer C, Kühn H, Borchert A. Molecular biology of glutathione peroxidase 4: from genomic structure to developmental expression and neural function. Biol Chem. 2007 oct;388(10):1007–17.

[135] Schreiber A, Stengel F, Zhang Z, Enchev RI, Kong EH, Morris EP, et al. Structural basis for the subunit assembly of the anaphase-promoting complex. Nature. 2011 feb;470(7333):227–32.

[136] Shen RF, Tai HH. Thromboxanes: synthase and receptors. J Biomed Sci. 1998 jan;5(3):153–72.

[137] Shinohara H, Yasuda T, Yamanashi Y. Dok-1 tyrosine residues at 336 and 340 are essential for the negative regulation of Ras-Erk signalling , but dispensable for. Genes Cells. 2004;2:601– 607.

[138] Shirayama M, Zachariae W, Ciosk R, Nasmyth K. The Polo-like kinase Cdc5p and the WD-repeat protein Cdc20p/fizzy are regulators and substrates of the anaphase promoting complex in Saccharomyces cerevisiae. EMBO J. 1998 mar;17(5):1336–49.

[139] Shuai K, Liu B. Regulation of JAK-STAT signalling in the immune system. Nat Rev Immunol. 2003 nov;3(11):900–11.

[140] Simister PC, Feller SM. Order and disorder in large multi-site docking proteins of the Gab family–implications for signalling complex formation and inhibitor design strategies. Mol Biosyst. 2012 jan;8(1):33–46.

[141] Singh P, Wang B, Maeda T, Palczewski K, Tesmer JJG. Structures of rhodopsin kinase in different ligand states reveal key elements involved in G protein-coupled receptor kinase activation. J Biol Chem. 2008 may;283(20):14053–62.

[142] Smith SO. Structure and activation of the visual pigment rhodopsin. Annu Rev Biophys. 2010 jun;39:309–28.

[143] Smrcka AV. G protein betagamma subunits: central mediators of G protein-coupled receptor signaling. Cell Mol Life Sci. 2008 jul;65(14):2191–214.

[144] Song MS, Salmena L, Pandolfi PP. The functions and regulation of the PTEN tumour suppressor. Nat Rev Mol Cell Biol. 2012 apr;13(5):283–296.

[145] Staal FJT, Clevers HC. Wnt signaling in the thymus. Curr Opin Immunol. 2003 apr;15(2):204–8.

[146] Stefan MI, Edelstein SJ, Le Novère N. An allosteric model of calmodulin explains differential activation of PP2B and CaMKII. Proc Natl Acad Sci U S A. 2008 aug;105(31):10768–73.

[147] Stennicke HR, Deveraux QL, Humke EW, Reed JC, Dixit VM, Salvesen GS. Caspase-9 can be activated without proteolytic processing. J Biol Chem. 1999 mar;274(13):8359–62.

[148] Stratton HF, Zhou J, Reed SI, Stone DE. The mating-specific G(alpha) protein of Saccharomyces cerevisiae downregulates the mating signal by a mechanism that is dependent on pheromone and independent of G(beta)(gamma) sequestration. Mol Cell Biol. 1996 nov;16(11):6325–37.

[149] Suzuki Y, Nakabayashi Y, Takahashi R. Ubiquitin-protein ligase activity of X-linked inhibitor of apoptosis protein promotes proteasomal degradation of caspase-3 and enhances its anti-apoptotic effect in Fas-induced cell death. Proc Natl Acad Sci U S A. 2001 jul;98(15):8662–7.

[150] Swift S, Leger AJ, Talavera J, Zhang L, Bohm A, Kuliopulos A. Role of the PAR1 receptor 8th helix in signaling: the 7-8-1 receptor activation mechanism. J Biol Chem. 2006 feb;281(7):4109–16.

[151] Traverso EE, Baskerville C, Liu Y, Shou W, James P, Deshaies RJ, et al. Characterization of the Net1 cell cycle-dependent regulator of the Cdc14 phosphatase from budding yeast. J Biol Chem. 2001 jun;276(24):21924–31.

[152] Uhlmann F, Wernic D, Poupart MA, Koonin EV, Nasmyth K. Cleavage of cohesin by the CD clan protease separin triggers anaphase in yeast. Cell. 2000 oct;103(3):375–86.

[153] Vadas O, Burke JE, Zhang X, Berndt A, Williams RL. Structural basis for activation and inhibition of class I phosphoinositide 3-kinases. Sci Signal. 2011 oct;4(195):re2.

[154] Varghese JN, Moritz RL, Lou MZ, Van Donkelaar A, Ji H, Ivancic N, et al. Structure of the extracellular domains of the human interleukin-6 receptor alpha -chain. Proc Natl Acad Sci U S A. 2002 dec;99(25):15959–64.

[155] Viadiu H, Stemmann O, Kirschner MW, Walz T. Domain structure of separase and its binding to securin as determined by EM. Nat Struct Mol Biol. 2005 jun;12(6):552–3.

[156] Wall MA, Coleman DE, Lee E, Iaiguez-lluhi JA, Posner BA, Gilman AG, et al. The Structure of the G Protein Heterotrimer GialPlyz. Cell. 1995;83:1047–1058.

[157] Ward CW, Lawrence MC, Streltsov Va, Adams TE, McKern NM. The insulin and EGF receptor structures: new insights into ligand-induced receptor activation. Trends Biochem Sci. 2007 mar;32(3):129–37.

[158] Wäsch R, Cross FR. APC-dependent proteolysis of the mitotic cyclin Clb2 is essential for mitotic exit. Nature. 2002 aug;418(6897):556–62.

[159] Waters SB, Holt KH, Ross SE, Syu LJ, Guan KL, Saltiel aR, et al. Desensitization of Ras activation by a feedback disassociation of the SOS-Grb2 complex. J Biol Chem. 1995 sep;270(36):20883–6.

[160] Wehrman T, He X, Raab B, Dukipatti A, Blau H, Garcia KC. Structural and mechanistic insights into nerve growth factor interactions with the TrkA and p75 receptors. Neuron. 2007 jan;53(1):25–38.

[161] Weinreich M, Liang C, Chen HH, Stillman B. Binding of cyclin-dependent kinases to ORC and Cdc6p regulates the chromosome replication cycle. Proc Natl Acad Sci U S A. 2001 sep;98(20):11211–7.

[162] Wilmes GM, Archambault V, Austin RJ, Jacobson MD, Bell SP, Cross FR. Interaction of the S-phase cyclin Clb5 with an "RXL" docking sequence in the initiator protein Orc6 provides an origin-localized replication control switch. Genes Dev. 2004 may;18(9):981–91.

[163] Winters MJ, Pryciak PM. Interaction with the SH3 domain protein Bem1 regulates signaling by the Saccharomyces cerevisiae p21-activated kinase Ste20. Mol Cell Biol. 2005 mar;25(6):2177–90.

[164] Wolgemuth DJ. Function of cyclins in regulating the mitotic and meiotic cell cycles in male germ cells. Cell Cycle. 2008;7:3509–3513.

[165] Won AP, Garbarino JE, Lim WA. Recruitment interactions can override catalytic interactions in determining the functional identity of a protein kinase. Proc Natl Acad Sci U S A. 2011 jun;108(24):9809–14.

[166] Xiao B, Spencer J, Clements A, Ali-khan N, Mittnacht S, Brocen C, et al. Crystal structure of the retinoblastoma tumor suppressor protein bound to E2F and the. Proc Natl Acad Sci U S A. 2003;100(5):2363–2368.

[167] Yamada T, Komoto J, Watanabe K, Ohmiya Y, Takusagawa F. Crystal Structure and Possible Catalytic Mechanism of Microsomal Prostaglandin E Synthase Type 2 (mPGES-2). Structure. 2005;2:1163–1176.

[168] Zähringer H, Burgert M, Holzer H, Nwaka S. Neutral trehalase Nth1p of Saccharomyces cerevisiae encoded by the NTH1 gene is a multiple stress responsive protein. FEBS Lett. 1997 aug;412(3):615–20.

[169] Zoncu R, Efeyan A, Sabatini DM. mTOR: from growth signal integration to cancer, diabetes and ageing. Nat Rev Mol Cell Biol. 2011 jan;12(1):21–35.

[170] van Es JH, Kirkpatrick C, van de Wetering M, Molenaar M, Miles A, Kuipers J, et al. Identification of APC2, a homologue of the adenomatous polyposis coli tumour suppressor. Curr Biol. 1999 jan;9(2):105–8.
