# Supplementary material for: Selection on Network Dynamics Drives Differential Rates of Protein Domain Evolution
Source: PLoS Genet. 2016 Jul 5;12(7):e1006132. doi: 10.1371/journal.pgen.1006132 (PMC4933380; doi:10.1371/journal.pgen.1006132)
Supplement: S1 Table — Spearman rank (ρ) and rank biserial (rb) correlation coefficients for variables evolutionary rate dN/dS (ω), dynamical influence (D), expression breadth (B), expression level (X), interaction degree (d), interaction betweenness centrality (C), and knock-out essentiality (E). Domains with missing values for any correlate were dropped prior to calculating correlations, and N represents the number of domains used in the analysis. For correlations, p-values were calculated via permutation of the data as described in the main text. For partial correlations, p-values were calculated by permutation of the residuals from the linear models [103, 104]. (PDF) [file pgen.1006132.s002.pdf]

Model	$\rho_{\omega,D}$ (p-val, N)	$\rho_{\omega,B}$ (p-val, N)	$\rho_{\omega,X}$ (p-val, N)	$\rho_{\omega,d}$ (p-val, N)	$\rho_{\omega,C}$ (p-val, N)	$rb_{\omega,E}$ (p-val, N)	$\rho_{D,B}$ (p-val, N)	$\rho_{D,X}$ (p-val, N)	$\rho_{D,d}$ (p-val, N)	$\rho_{D,C}$ (p-val, N)	$rb_{D,E}$ (p-val, N)	$\rho_{\omega,D\|B,X,d,C,E,Gr}$ (p-val, N)
EGF/NGF signaling [28]	-0.56 (0.0054, 32)	-0.60 (0.0040, 28)	-0.51 (0.0244, 28)	-0.22 (0.3332, 32)	-0.21 (0.3679, 32)	-0.34 (0.4698, 31)	+0.55 (0.0045, 28)	+0.31 (0.1385, 28)	+0.03 (0.8579, 32)	+0.08 (0.6577, 32)	+0.84 (0.0150, 31)	-0.30 (0.1284, 27)
Arachadonic acid signaling [29]	-0.54 (0.1140, 11)	+0.01 (0.9938, 10)	-0.12 (0.7878, 10)	-0.35 (0.3857, 10)	-0.35 (0.3750, 10)	+0.43 (0.3748, 10)	+0.72 (0.0597, 10)	+0.82 (0.0200, 10)	+0.10 (0.8110, 10)	-0.16 (0.7295, 10)	-0.05 (0.9363, 10)	+0.39 (0.3142, 9)
EGF/NGF signaling [30]	-0.29 (0.1633, 39)	-0.45 (0.0425, 33)	-0.39 (0.0932, 33)	-0.08 (0.7266, 39)	-0.09 (0.6664, 39)	-0.73 (0.0286, 38)	+0.05 (0.7906, 33)	-0.01 (0.9460, 33)	+0.36 (0.0325, 39)	+0.38 (0.0252, 39)	+0.13 (0.6652, 38)	-0.33 (0.0620, 33)
EGF/MAPK cascade [31]	-0.35 (0.1217, 20)	-0.30 (0.2565, 20)	-0.32 (0.2346, 20)	-0.12 (0.6499, 20)	-0.16 (0.5635, 20)	+0.16 (0.7951, 18)	+0.34 (0.1422, 20)	+0.36 (0.1146, 20)	+0.16 (0.4896, 20)	+0.17 (0.4846, 20)	-0.38 (0.3062, 18)	-0.13 (0.5870, 18)
Rho-kinase activation [32]	-0.23 (0.1619, 30)	-0.15 (0.5931, 28)	+0.05 (0.8565, 28)	-0.46 (0.0383, 30)	-0.40 (0.0905, 30)	+0.29 (0.4141, 24)	-0.25 (0.2568, 28)	-0.26 (0.2353, 28)	+0.12 (0.5621, 30)	+0.00 (0.9889, 30)	+0.44 (0.1260, 24)	-0.37 (0.0731, 24)
Extrinsic apoptosis [33]	-0.27 (0.2618, 29)	-0.27 (0.3801, 26)	-0.06 (0.8549, 26)	+0.10 (0.7261, 29)	+0.15 (0.6218, 29)	+0.04 (0.9223, 28)	+0.17 (0.5036, 26)	-0.28 (0.2700, 26)	+0.05 (0.8332, 29)	-0.04 (0.8682, 29)	-0.30 (0.3457, 28)	-0.04 (0.8654, 25)
EGF/Insulin crosstalk [34]	-0.25 (0.1675, 43)	-0.19 (0.4154, 42)	-0.20 (0.3925, 42)	-0.04 (0.8584, 43)	-0.07 (0.7684, 43)	-0.46 (0.2218, 43)	+0.40 (0.0178, 42)	+0.21 (0.2136, 42)	+0.01 (0.9392, 43)	+0.03 (0.8830, 43)	+0.55 (0.0589, 43)	-0.16 (0.3311, 42)
G1 cell cycle progression [35]	-0.24 (0.5880, 15)	-0.10 (0.8366, 14)	+0.18 (0.6817, 14)	-0.51 (0.1556, 15)	-0.43 (0.2761, 15)	-0.23 (0.7689, 14)	+0.22 (0.5168, 14)	-0.06 (0.8551, 14)	+0.59 (0.0469, 15)	+0.58 (0.0504, 15)	+0.38 (0.6106, 14)	+0.20 (0.4844, 14)
ErbB signaling [36]	-0.20 (0.2496, 41)	-0.21 (0.2556, 38)	-0.07 (0.7019, 38)	-0.24 (0.1737, 41)	-0.19 (0.2900, 41)	-0.19 (0.5382, 39)	+0.09 (0.5785, 38)	+0.08 (0.6370, 38)	-0.07 (0.6594, 41)	+0.00 (0.9909, 41)	-0.45 (0.0996, 39)	-0.29 (0.0887, 36)
Wnt/Erk crosstalk [37]	-0.08 (0.8021, 15)	-0.28 (0.4417, 15)	-0.28 (0.4524, 15)	-0.32 (0.3879, 15)	-0.47 (0.1798, 15)	+0.45 (0.6079, 12)	-0.27 (0.3317, 15)	-0.48 (0.0679, 15)	-0.07 (0.8105, 15)	-0.27 (0.3282, 15)	+1.00 (0.1253, 12)	+0.62 (0.0300, 12)
Rod phototransduction [38]	+0.42 (0.2028, 19)	+0.10 (0.7304, 17)	+0.46 (0.0869, 17)	+0.11 (0.6947, 19)	+0.10 (0.7135, 19)		+0.09 (0.7753, 17)	+0.26 (0.4078, 17)	+0.37 (0.1989, 19)	+0.26 (0.3731, 19)		+0.17 (0.5606, 15)
IL-6 signaling [39]	+0.45 (0.0928, 26)	-0.20 (0.4933, 25)	-0.32 (0.2795, 25)	-0.45 (0.0907, 26)	-0.36 (0.1949, 26)	+0.00 (1.0000, 26)	+0.02 (0.9198, 25)	+0.12 (0.5772, 25)	-0.19 (0.3361, 26)	-0.14 (0.4833, 26)	-0.06 (0.8465, 26)	+0.43 (0.0310, 25)
