# Supplementary material for: Selection on Network Dynamics Drives Differential Rates of Protein Domain Evolution
Source: PLoS Genet. 2016 Jul 5;12(7):e1006132. doi: 10.1371/journal.pgen.1006132 (PMC4933380; doi:10.1371/journal.pgen.1006132)
Supplement: S2 Table — As in S1 Table, but for yeast models, which have knock-out growth rate (Gr) data. (PDF) [file pgen.1006132.s003.pdf]

Model	$\rho_{\omega,D}$ (p-val, N)	$\rho_{\omega,X}$ (p-val, N)	$\rho_{\omega,d}$ (p-val, N)	$\rho_{\omega,C}$ (p-val, N)	$rb_{\omega,E}$ (p-val, N)	$\rho_{\omega,Gr}$ (p-val, N)	$\rho_{D,X}$ (p-val, N)	$\rho_{D,d}$ (p-val, N)	$\rho_{D,C}$ (p-val, N)	$rb_{D,E}$ (p-val, N)	$\rho_{D,Gr}$ (p-val, N)	$\rho_{\omega,D\|B,X,d,C,E,Gr}$ (p-val, N)
Trehalose biosynthesis [40]	-0.75 (0.0718, 7)	+0.32 (0.5030, 7)	-0.56 (0.3729, 5)	-0.90 (0.0789, 5)	+0.60 (0.2871, 7)	-0.05 (0.9144, 7)	-0.14 (0.7851, 7)	+0.56 (0.3652, 5)	+0.80 (0.1299, 5)	-0.20 (0.8545, 7)	-0.34 (0.4536, 7)	-0.76 (0.1338, 5)
Glycolysis [41]	-0.41 (0.0958, 18)	+0.85 (<0.0001, 18)	+0.33 (0.1960, 17)	+0.04 (0.8727, 17)	+0.12 (0.7255, 18)	-0.22 (0.3815, 18)	-0.33 (0.1818, 18)	-0.20 (0.4458, 17)	-0.02 (0.9241, 17)	+0.14 (0.6600, 18)	-0.03 (0.9019, 18)	-0.11 (0.6842, 17)
Cell cycle regulation [42]	-0.41 (0.0207, 29)	-0.32 (0.1011, 29)	-0.44 (0.0242, 29)	-0.21 (0.2984, 29)	-0.08 (0.7448, 29)	-0.00 (0.9922, 29)	+0.16 (0.3987, 29)	+0.07 (0.7299, 29)	-0.16 (0.3951, 29)	+0.17 (0.4408, 29)	-0.14 (0.4569, 29)	-0.40 (0.0314, 29)
Mitotic exit [43]	-0.36 (0.2476, 17)	-0.47 (0.0577, 17)	-0.42 (0.0988, 17)	-0.16 (0.5568, 17)	+0.35 (0.4011, 17)	-0.26 (0.3208, 17)	+0.26 (0.2874, 17)	+0.22 (0.3841, 17)	+0.02 (0.9445, 17)	-0.73 (0.0434, 17)	+0.52 (0.0268, 17)	+0.04 (0.8872, 17)
Mitotic exit [44]	-0.31 (0.1362, 27)	-0.28 (0.2110, 27)	-0.40 (0.0609, 27)	-0.16 (0.4605, 27)	-0.12 (0.6463, 27)	+0.05 (0.8410, 27)	+0.25 (0.1995, 27)	+0.01 (0.9465, 27)	-0.14 (0.4952, 27)	-0.08 (0.7424, 27)	+0.12 (0.5388, 27)	-0.29 (0.1492, 27)
Pheromone pathway [45]	-0.09 (0.6949, 23)	-0.21 (0.4152, 23)	-0.17 (0.5010, 23)	-0.28 (0.2547, 23)	-0.27 (0.3918, 23)	+0.23 (0.3544, 23)	+0.40 (0.0614, 23)	-0.28 (0.2044, 23)	+0.16 (0.4589, 23)	-0.37 (0.1615, 23)	+0.36 (0.0980, 23)	-0.21 (0.3222, 23)
