# Supplementary material for: Selection on Network Dynamics Drives Differential Rates of Protein Domain Evolution
Source: PLoS Genet. 2016 Jul 5;12(7):e1006132. doi: 10.1371/journal.pgen.1006132 (PMC4933380; doi:10.1371/journal.pgen.1006132)
Supplement: S3 Table — For each pair of models with at least four overlapping domains, shown is the Spearman rank correlation and number of overlapping domains. (PDF) [file pgen.1006132.s004.pdf]

	EGF/NGF signaling [30]	EGF/MAPK cascade [31]	EGF/Insulin crosstalk [34]	ErbB signaling [36]	IL-6 signaling [39]	Mitotic exit [43]	Mitotic exit [44]
EGF/NGF signaling [28]	+0.12, 23	+0.13, 12	+0.86, 13	+0.46, 14	+0.17, 10		
EGF/NGF signaling [30]		+0.13, 16	+0.08, 17	+0.32, 19	+0.30, 10		
EGF/MAPK cascade [31]			-0.05, 11	-0.00, 18	+0.54, 10		
EGF/Insulin crosstalk [34]				+0.00, 19	-0.39, 10		
ErbB signaling [36]					-0.36, 9		
Cell cycle regulation [42]						+0.52, 15	+0.63, 22
Mitotic exit [43]							+0.79, 17
