# Supplementary material for: Selection on Network Dynamics Drives Differential Rates of Protein Domain Evolution
Source: PLoS Genet. 2016 Jul 5;12(7):e1006132. doi: 10.1371/journal.pgen.1006132 (PMC4933380; doi:10.1371/journal.pgen.1006132)
Supplement: S4 Table — Dynamical influence was recalculated using finite perturbations ΔyΔk=y(k+)-y(k-)k+-k- instead of differential perturbations dydk (Eq 1), with k+ and k− being ±25% perturbations of each parameter. Tabulated are the rank correlations between domain influences calculated using finite and differential perturbations. The almost perfect correlations suggest that our dynamical influence analysis also applies to mutations of moderate effect. (PDF) [file pgen.1006132.s005.pdf]

Model	correlation
EGF/NGF signaling [28]	1.00
Arachadonic acid signaling [29]	1.00
EGF/NGF signaling [30]	1.00
EGF/MAPK cascade [31]	1.00
Rho-kinase activation [32]	0.91
Extrinsic apoptosis [33]	1.00
EGF/Insulin crosstalk [34]	0.92
G1 cell cycle progression [35]	1.00
ErbB signaling [36]	0.99
Wnt/Erk crosstalk [37]	0.96
Rod phototransduction [38]	1.00
IL-6 signaling [39]	1.00
Trehalose biosynthesis [40]	1.00
Glycolysis [41]	0.98
Cell cycle regulation [42]	1.00
Mitotic exit [43]	0.99
Mitotic exit [44]	1.00
Pheromone pathway [45]	1.00
