# Supplementary material for: Selection on Network Dynamics Drives Differential Rates of Protein Domain Evolution
Source: PLoS Genet. 2016 Jul 5;12(7):e1006132. doi: 10.1371/journal.pgen.1006132 (PMC4933380; doi:10.1371/journal.pgen.1006132)
Supplement: S5 Table — Meta-analysis confidence intervals and permutation p-values as in Table 1. Spearman rank (ρ) correlation coefficients for variables evolutionary rate dN/dS (ω), dynamical influence (D), model-derived expression (MX), model-derived interaction degree (Md), model-derived interaction centrality (MC), expression breadth (B), knock-out essentiality (E), and knock-out growth rate (Gr). (PDF) [file pgen.1006132.s006.pdf]

	population mean correlation (95% C.I.)	permutation p-value
$\rho_{\omega,MX}$	-0.17 (-0.28, -0.05)	0.0090
$\rho_{\omega,Md}$	-0.09 (-0.22, +0.05)	0.1841
$\rho_{\omega,MC}$	-0.16 (-0.29, -0.03)	0.0093
$\rho_{D,MX}$	+0.08 (-0.04, +0.21)	0.1335
$\rho_{D,Md}$	-0.07 (-0.16, +0.01)	0.1792
$\rho_{D,MC}$	-0.01 (-0.10, +0.07)	0.8109
$\rho_{\omega,D\|B,MX,Md,MC,E,Gr}$	-0.20 (-0.33, -0.07)	0.0003
