# Supplementary material for: Selection on Network Dynamics Drives Differential Rates of Protein Domain Evolution
Source: PLoS Genet. 2016 Jul 5;12(7):e1006132. doi: 10.1371/journal.pgen.1006132 (PMC4933380; doi:10.1371/journal.pgen.1006132)
Supplement: S6 Table — Meta-analysis mean correlations and permutation p-values as in Table 1, but without overlapping domains between models. Values are the 50th (5th, 95th) quantiles of correlations and p-values calculated from 1000 runs in which each domain that appears in multiple models was considered for only a single randomly chosen model. (PDF) [file pgen.1006132.s007.pdf]

	correlation	p-value
$\rho_{\omega,D}$	-0.22 (-0.25, -0.18)	0.0010 (<0.0001, 0.0073)
$\rho_{\omega,B}$	-0.22 (-0.25, -0.19)	0.0183 (0.0063, 0.0449)
$\rho_{\omega,X}$	-0.06 (-0.09, -0.03)	0.4081 (0.2549, 0.6628)
$\rho_{\omega,d}$	-0.20 (-0.23, -0.18)	0.0043 (0.0015, 0.0129)
$\rho_{\omega,C}$	-0.20 (-0.23, -0.17)	0.0059 (0.0014, 0.0173)
$rb_{\omega,E}$	-0.06 (-0.12, -0.01)	0.5790 (0.3158, 0.9061)
$\rho_{\omega,Gr}$	+0.02 (-0.05, +0.11)	0.7825 (0.3377, 0.9681)
$\rho_{D,B}$	+0.16 (+0.10, +0.22)	0.0479 (0.0066, 0.2275)
$\rho_{D,X}$	+0.07 (+0.03, +0.11)	0.3112 (0.1007, 0.7019)
$\rho_{D,d}$	+0.10 (+0.05, +0.13)	0.1391 (0.0390, 0.4178)
$\rho_{D,C}$	+0.10 (+0.05, +0.14)	0.1355 (0.0329, 0.4177)
$rb_{D,E}$	+0.07 (+0.02, +0.14)	0.4893 (0.1868, 0.8023)
$\rho_{D,Gr}$	+0.04 (-0.04, +0.13)	0.7049 (0.2695, 0.9776)
$\rho_{\omega,D\|B,X,d,C,E,Gr}$	-0.15 (-0.21, -0.08)	0.0206 (0.0011, 0.2278)
