# Supplementary figures and images for: Selection on Network Dynamics Drives Differential Rates of Protein Domain Evolution
Source: PLoS Genet. 2016 Jul 5;12(7):e1006132. doi: 10.1371/journal.pgen.1006132 (PMC4933380; doi:10.1371/journal.pgen.1006132)

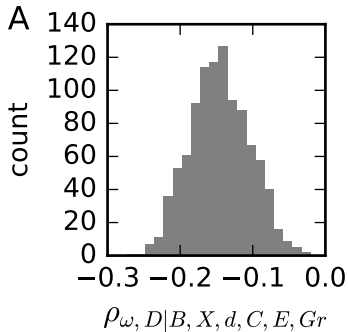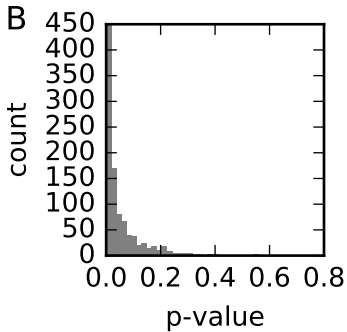

Supplement: S1 Fig — Shown are the full distributions that are summarized in the last row of S6 Table. A: The partial correlation between domain dynamical influence and evolutionary rate is negative for all randomizations. B: The distribution of p-values is strongly concentrated below 0.05. The tail of larger p-values is generated by randomizations that concentrate domains in the few models with a positive correlation. (PDF) [file pgen.1006132.s008.pdf]

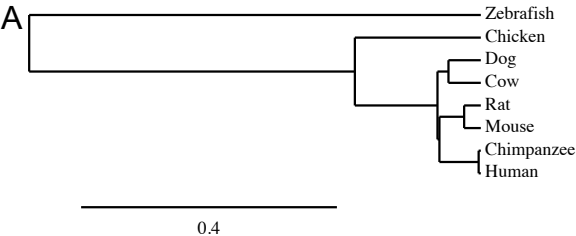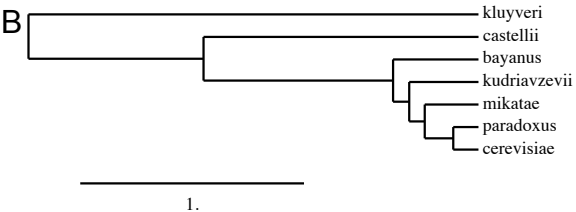

Supplement: S2 Fig — A: Vertebrates. B: Yeasts. In both, branch lengths represent amino acid divergence. (PDF) [file pgen.1006132.s009.pdf]
